# Tau association with synaptic vesicles causes presynaptic dysfunction

**DOI:** 10.1038/ncomms15295

**Published:** 2017-05-11

**Authors:** Lujia Zhou, Joseph McInnes, Keimpe Wierda, Matthew Holt, Abigail G. Herrmann, Rosemary J. Jackson, Yu-Chun Wang, Jef Swerts, Jelle Beyens, Katarzyna Miskiewicz, Sven Vilain, Ilse Dewachter, Diederik Moechars, Bart De Strooper, Tara L. Spires-Jones, Joris De Wit, Patrik Verstreken

**Affiliations:** 1 VIB-KU Leuven Center for Brain & Disease Research, Leuven 3000, Belgium; 2 KU Leuven, Department of Neurosciences, Leuven Research Institute for Neuroscience and Disease (LIND), Leuven 3000, Belgium; 3 University of Edinburgh, Centre for Cognitive and Neural Systems, Center for Dementia Prevention and Euan MacDonald Centre, Edinburgh EH8 9JZ, UK; 4 Catholic University of Louvain, Alzheimer Dementia Group, Institute of Neuroscience, Brussels 1200, Belgium; 5 University of Hasselt, Biomedical Research Institute, Hasselt 3500, Belgium; 6 A Division of Janssen Pharmaceutica NV, Neuroscience Department, Janssen Research and Development, Beerse 2340, Belgium

## Abstract

Tau is implicated in more than 20 neurodegenerative diseases, including Alzheimer's disease. Under pathological conditions, Tau dissociates from axonal microtubules and missorts to pre- and postsynaptic terminals. Patients suffer from early synaptic dysfunction prior to Tau aggregate formation, but the underlying mechanism is unclear. Here we show that pathogenic Tau binds to synaptic vesicles via its N-terminal domain and interferes with presynaptic functions, including synaptic vesicle mobility and release rate, lowering neurotransmission in fly and rat neurons. Pathological Tau mutants lacking the vesicle binding domain still localize to the presynaptic compartment but do not impair synaptic function in fly neurons. Moreover, an exogenously applied membrane-permeable peptide that competes for Tau-vesicle binding suppresses Tau-induced synaptic toxicity in rat neurons. Our work uncovers a presynaptic role of Tau that may be part of the early pathology in various Tauopathies and could be exploited therapeutically.

## Abstract (web summary)

Mislocalisation of tau occurs in several neurodegenerative diseases and is thought to contribute to synaptic function. The authors show that presynaptically, tau binds to synaptic vesicles via the N-terminus which contributes to synaptic dysfunction.

Accumulation of abnormal Tau in human brain has been implicated in major neurodegenerative diseases termed Tauopathies, including Alzheimer's disease (AD), Parkinson disease (PD), and Frontotemporal dementia with Parkinsonsim-17 (FTDP-17) (reviewed in refs 1, 2, 3). More than 40 mutations in the Tau-encoding *MAPT* locus have been identified as a direct cause for FTDP-17, and slightly higher expression of Tau driven by the Tau H1 haplotype increases risk for AD and PD^4^,^5^,^6^. In addition, several groups have shown that loss of Tau prevents Aβ and other excitotoxin-induced neuronal toxicity and cognitive deficits in mouse models of AD and other neurodegenerative diseases, suggesting that Tau is a direct mediator of neurodegenerative disease^7^,^8^,^9^,^10^,^11^,^12^.

Synaptic dysfunction is thought to be an early pathological manifestation in AD and other Tauopathies^13^,^14^,^15^,^16^. Recent studies suggest that soluble Tau, rather than the neurofibrillary tangle-associated aggregated form, is the main toxic element that is able to induce early synaptic deficits preceding synapse and neuronal loss^17^,^18^,^19^,^20^,^21^,^22^,^23^,^24^. However, pathogenic mechanisms of non-aggregated Tau in neurons remain enigmatic.

Tau is mainly expressed in neurons and is highly concentrated in the axon, where it associates with microtubules. However, under disease conditions, Tau detaches from microtubules as abnormal phosphorylation or FTDP-17 pathogenic mutations lower its binding affinity for microtubules^3^,^25^. Pathological Tau has been detected to colocalize with both pre- and post-synaptic markers in the staining of isolated synaptosomes from AD patient brains^26^,^27^,^28^,^29^. Immunohistological analyses of transgenic mouse brains made similar observations, that FTDP-17 pathogenic mutations drive mislocalization of Tau to both presynaptic terminals and dendritic spines^30^,^31^. These data together implicate that Tau missorts to both pre- and post- synapses under pathological conditions. Several studies have suggested a pathophysiological role of Tau at dendritic spines in affecting the trafficking of postsynaptic receptors^21^,^32^. In contrast, the role for mis-localized Tau at presynaptic terminals remains unclear.

In this study, we show that FTDP-17 mutant Tau mislocalizes to presynaptic terminals in fly neurons, where it binds synaptic vesicles to elicit presynaptic dysfunction. The underlying mechanism involves a dual action of Tau in binding synaptic vesicles and simultaneously promoting presynaptic actin polymerization to crosslink vesicles and restrict their mobilization. Pathogenic Tau thus reduces synaptic transmission during prolonged neuronal activity in fly neurons, and similar defects are observed in rat neurons. Disruption of the interaction between Tau and synaptic vesicles is sufficient to rescue these defects, suggesting that the mode-of-action we identified is a key mechanism for Tau-induced presynaptic pathology.

## Results

### Pathogenic Tau binds to vesicles at presynaptic terminals

Pathological Tau was previously detected at both pre- and post-synaptic compartments in isolated synaptosomes from AD patient brains^26^,^28^,^29^. We applied array tomography to determine the localization of pathological Tau *in situ* in the brain of AD patients. Ultrathin (70 nm) brain sections were prepared and labelled with antibodies against pathological phospho-Tau (AT8) and the presynaptic marker protein Synapsin. Our data shows that pathological phospho-Tau is present at presynaptic terminals of AD patient brains but is absent from the presynaptic terminals of healthy controls (Supplementary Fig. 1). Our data, together with previous studies, argues for the relevance of exploring the role of presynaptic Tau in eliciting synaptic pathology.

Given that the mechanisms of presynaptic function are well conserved across species^33^,^34^, we resorted to *Drosophila* larval neuromuscular junctions (NMJs) to study the presynaptic role of Tau. Presynaptic terminals at the NMJ are large, allowing for imaging, and they can be genetically separated from the post-synaptic muscle. We inserted expression constructs of human wild type or FTDP-17 pathogenic mutant Tau (R406W, V337M and P301L, 0N4R isoform) at identical genomic loci in the fly genome, resulting in very similar expression levels (Supplementary Fig. 2a,b). We compared Tau expression to previously published lines^35^,^36^,^37^,^38^ and find our new constructs result in about four times lower expression levels (Supplementary Fig. 2c,d). Furthermore, the expression level of human Tau in fly brains is in a similar range of the level of Tau in human brain samples (Supplementary Fig. 2e,f). Our new lines are thus optimized to express Tau at a low level to recapitulate Tau expression in human brains.

**Figure 1:**
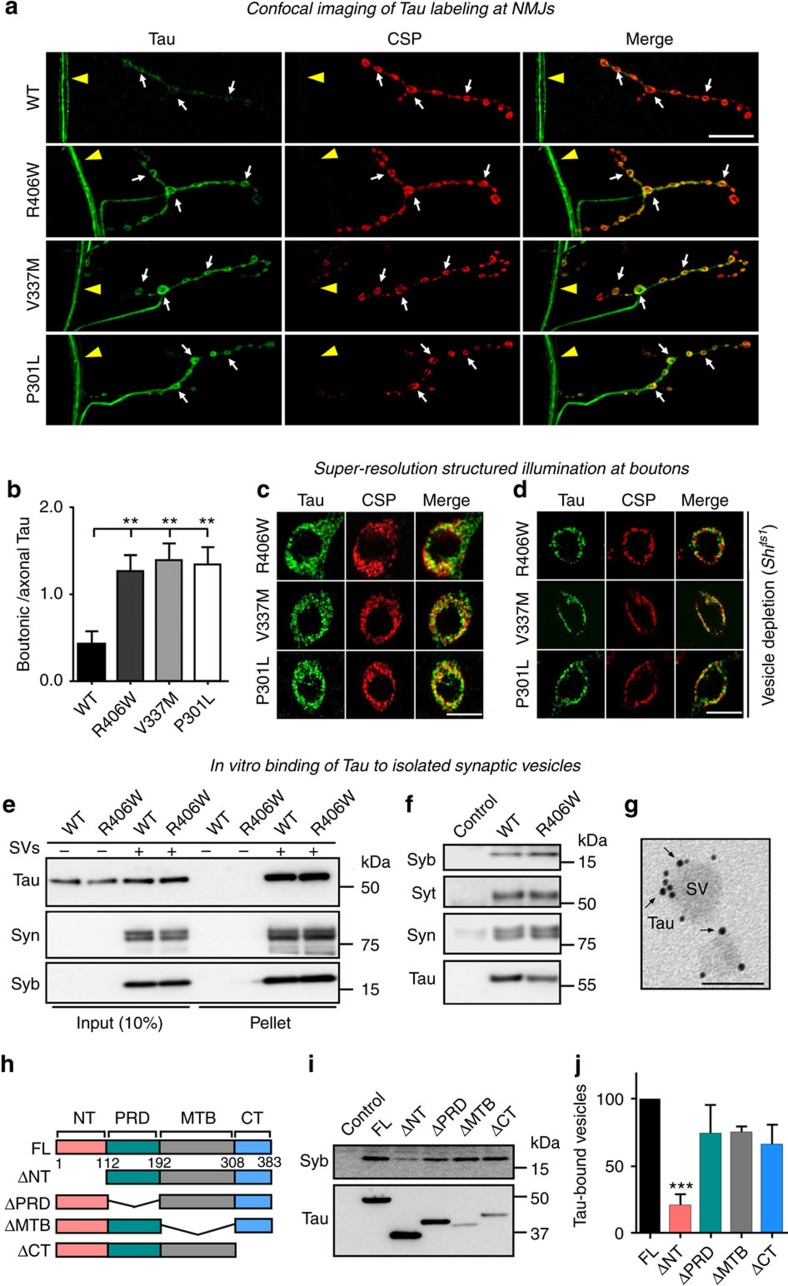
Tau localizes to presynaptic terminals and binds to synaptic vesicles via its N-terminal domain. (**a**) Tau and CSP immunolabeling at neuromuscular junctions (NMJs) of *Drosophila* larvae expressing WT or FTDP-17 pathogenic mutant Tau (R406W, V337M or P301L) under the D42-Gal4 motor neuron driver. Axons (arrowheads) and synaptic boutons (arrows) are indicated. Scale bar, 20 μm. (**b**) Quantification of fluorescence intensity of Tau within synaptic boutons (SBs) as ratio to the intensity of axonal Tau. One-way ANOVA, **P=0.0030, 0.0019, 0.0041 (R406W, V337M, P301L) *n*=10 (R406W, V337M, P301L) or 12 (WT) NMJs from 5 to 6 animals. Data present mean±s.e.m. (**c**,**d**) Super-resolution structured illumination microscopy analysis of Tau and CSP immunolabeling within SBs under non-treated condition (**c**) or after depletion of synaptic vesicles in *Shi*^*ts1*^ mutant background by KCl stimulation at the non-permissive temperature (**d**). Scale bar, 5 μm. (**e**,**f**) Immunoblots of Tau (anti-His tag) and synaptic vesicle (SV) proteins Synaptobrevin (Syb), Synaptotagmin (Syt) and Synapsin (Syn) from sedimentation assay (**e**) and co-immunoprecipitation (co-IP) using anti-His antibodies (**f**) assessing recombinant human Tau binding to purified synaptic vesicles. (**g**) Electron microscopy imaging of recombinant Tau (probed by Ni-NTA-Nanogold) bound to ultrapure synaptic vesicles *in vitro*. Scale bar, 50 nm. (**h**–**j**) Mapping of the vesicle-binding domain of Tau *in vitro* by co-IP assay. Truncations of the N-terminal (NT), proline-rich (PRD), microtubule-binding (MTB) or C-terminal (CT) domains of Tau were generated as indicated in the schematic (**h**). Immunoblots of recombinant Tau domain-truncations and Syb (SV marker) from co-IP using anti-His antibodies (**i**) and quantification of relative Syb intensity (**j**). One-way ANOVA, ***P=0.002, *n*=3 independent experiments.

We then assessed Tau localization in fly neurons. In motor neurons, both wild type and pathogenic mutant Tau are localized to axons. However, compared to wild type Tau, the pathogenic mutants show increased localization to presynaptic terminals labelled by the synaptic vesicle marker CSP (Fig. 1a,b). The increased presynaptic localization of the pathogenic mutant Tau is likely attributed to their lower affinity for binding microtubules, as previously reported^3^,^25^ and parallels our observations based on array tomography in human patient brains, where we also observed pathological Tau at presynaptic terminals (Supplementary Fig. 1).

We next assessed the sub-boutonic localization and distribution of synaptic-localized Tau using super-resolution structured illumination microscopy. We find that pathogenic Tau at presynaptic boutons is distributed in a ‘doughnut-like' pattern that closely resembles that of synaptic vesicles marked by anti-CSP labelling (Fig. 1c). We therefore questioned whether Tau associates with synaptic vesicles and hereto performed a vesicle depletion assay using a temperature sensitive Dynamin mutant (*shi*^*ts1*^) that blocks endocytosis at restrictive temperature^39^. When stimulated with KCl, synaptic vesicles fuse with the membrane but are not retrieved by endocytosis, as shown by the re-localization of the synaptic vesicle marker CSP to the plasma membrane (Fig. 1d). Similar to CSP, Tau relocates to the plasma membrane (Fig. 1d), suggesting that mislocalized pathogenic Tau at presynaptic terminals associates with synaptic vesicles *in vivo*.

To analyse the vesicle-binding properties of Tau, we prepared ultrapure synaptic vesicles by controlled-pore glass chromatography from rat brain (Supplementary Fig. 3a–e) and incubated the vesicles with purified recombinant human Tau (Supplementary Fig. 3f–h). Recombinant Tau co-sediments with synaptic vesicles in a vesicle sedimentation assay (Fig. 1e). Synaptic vesicles also co-immunoprecipitate using recombinant Tau as bait, evidenced by the recovery of synaptic vesicle marker proteins (Fig. 1f). Finally, electron microscopy of recombinant Tau incubated with isolated synaptic vesicles *in vitro* shows the protein binds to vesicle membranes (Fig. 1g). Note that both wild type and pathogenic mutant Tau are able to bind to synaptic vesicles *in vitro* (Fig. 1e,f); however, only mutant Tau is prominently present at presynaptic terminals, allowing it to bind to synaptic vesicles *in vivo* (Fig. 1a,b).

To determine the mode of vesicle binding we generated and purified Tau-deletion proteins and assessed if they could co-immunoprecipitate synaptic vesicles. Only truncation of the N-terminus (aa_1–112_) of Tau, but not the truncation of any other domain, strongly impedes with the binding of Tau to synaptic vesicles (Fig. 1h–j). In agreement, the Tau N-terminal fragment alone co-immunoprecipitates vesicles (Supplementary Fig. 3i). Therefore, the N-terminus of Tau is necessary and sufficient for synaptic vesicle binding.

### Pathogenic Tau restricts synaptic vesicle mobilization

**Figure 2:**
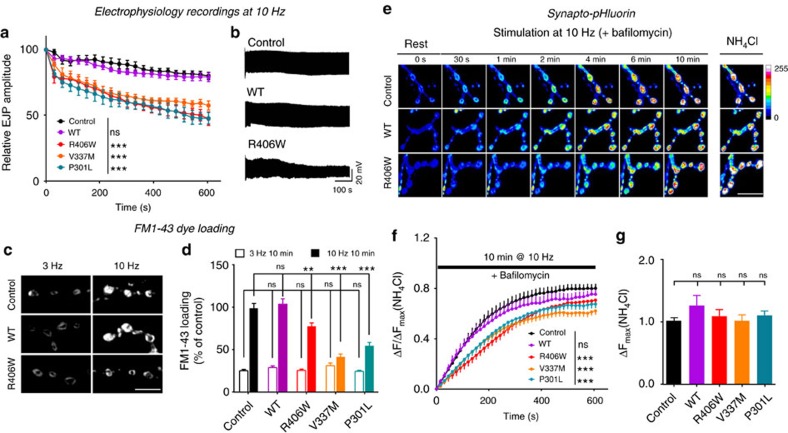
Pathogenic mutant Tau reduces synaptic transmission and vesicle cycling/release during sustained high-frequency stimulation. *Drosophila* larvae used in these assays express UAS-Tau (WT, R406W, V337M or P301L) under the D42-Gal4 motor neuron driver. (**a**,**b**) Electrophysiological recordings of synaptic transmission during 10 Hz stimulation for 10 min. Plot of evoked junction potential (EJP) amplitudes (**a**) and representative traces (**b**). Two-way ANOVA, ***P<0.0001, *n*=7 (Control, WT) or 9 (R406W, V337M, P301L) NMJs (animals). (**c**,**d**) FM1-43 dye loading with stimulation at 3 Hz (recycling vesicle pool) or 10 Hz (reserve vesicle pool) for 10 min. Representative images of FM1-43 dye loading (**c**) and plot of FM1-43 dye loading intensity (**d**). One-way ANOVA, **P=0.0028 (R406W), ***P=0.0001(V337M, P301L), *n*=14 (WT, R406W, V337M, P301L) or 20 (Control) NMJs (animals). Scale bar, 10 μm. (**e**–**g**) Synapto-pHluorin (SpH) responses to stimulation at 10 Hz with the presence of bafilomycin. Representative images of SpH responses (**e**) and plot of fluorescence change Δ*F* at ratio to maximal Δ*F* (NH_4_Cl dequenching) (**f**). Two-way ANOVA, ***P<0.0001, *n*=7 (WT, R406W, V337M, P301L) or 11 (Control) NMJs (animals). Plot of maximal ΔF (NH_4_Cl dequenching) calibrated to control levels (**g**). One-way ANOVA, ns, not significant. Scale bar, 20 μm. Data present mean±s.e.m.

Next we asked if vesicle-associated Tau elicits functional defects at presynaptic terminals. We recorded the spontaneous miniature excitatory junctional potentials (mEJPs) at fly NMJs and found that Tau-expressing animals do not show differences from control animals in mEJP frequency and mean amplitude (Supplementary Fig. 4a–d). We then stimulated motor neurons and recorded excitatory junctional potentials (EJPs). At low-frequency stimulation (0.2 Hz), the EJP amplitudes measured in controls and mutant Tau-expressing animals are similar (Supplementary Fig. 4e,f). However, during sustained high-frequency stimulation (10 Hz), pathogenic Tau mutant-expressing animals fail to maintain normal levels of release (Fig. 2a,b). Such a defect could reflect slowed synaptic vesicle reformation by endocytosis^40^,^41^,^42^, a reduced number of synaptic vesicles that participate in release, and/or slower vesicle mobilization^43^,^44^,^45^,^46^.

We first assessed synaptic vesicle endocytosis using SynaptopHluorin (SpH), a pH sensitive GFP fused to the luminal domain of the synaptic vesicle protein Synaptobrevin^47^. The fusion of synaptic vesicles with the presynaptic membrane causes SpH fluorescence to increase, while vesicle endocytosis and acidification results in a decrease in fluorescence. We did not observe significant differences in the rise and decay of SpH fluorescence in response to short stimulation paradigms in controls and mutant Tau-expressing animals (Supplementary Fig. 5). In an alternative approach, we also used an exogenously added fluorescent dye (FM 1–43) that is endocytosed into newly formed synaptic vesicles. During mild 3 Hz stimulation, we did not observe a difference in FM 1–43 uptake in the pathogenic Tau-expressing animals and in controls (Fig. 2c,d). Hence, under these conditions, endocytosis is not affected.

We next assessed synaptic vesicle mobilization during sustained stimulation. We measured SpH fluorescence during 10 min of 10 Hz stimulation in the presence of bafilomycin, a drug that blocks reacidification of newly endocytosed vesicles. Thus, vesicles that fuse at least once during the stimulation paradigm remain fluorescent, revealing the cumulative signals from vesicle fusion over time. Expression of pathogenic Tau mutants slows the vesicle release rate significantly, indicating a smaller active vesicle pool compared to controls or animals expressing wild type Tau (Fig. 2e,f). This effect is not due to a change in total synaptic vesicle pool size, as NH_4_Cl unquenching of all SpH fluorescence at the synapse does not show significant differences (Fig. 2g). We further confirmed the smaller cycling vesicle pool upon expression of pathogenic Tau by labelling the entire cycling vesicle pool with FM 1–43 dye using prolonged high frequency stimulation (10 min at 10 Hz). We observe significantly less dye uptake in pathogenic Tau, but not wild type Tau-expressing NMJs as compared to controls (Fig. 2c,d), indicating fewer labelled vesicles. Note that only mutant Tau, not wild type Tau elicits vesicle cycling defects, again correlating with the increased presynaptic localization of the mutant proteins (Fig. 1).

**Figure 3:**
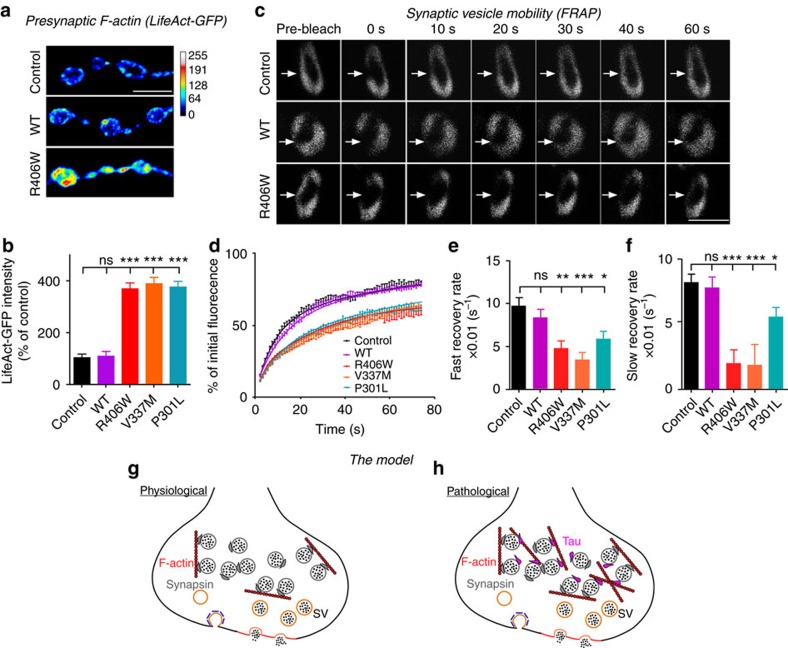
Pathogenic mutant Tau increases F-actin levels and reduces synaptic vesicle mobility at presynaptic terminals. *Drosophila* larvae used in these assays express UAS-Tau (WT, R406W, V337M or P301L) under the D42-Gal4 motor neuron driver. (**a**,**b**) Immunolabeling of LifeAct-GFP probing for F-actin within synaptic boutons. Representative images of immunolabeling (**a**) and quantification of LifeAct-GFP intensity (**b**). One-way ANOVA, ***P<0.0001, *n*=12 (WT, R406W, V337M, P301L) or 14 (Control) NMJs (two NMJs per animal). Scale bar, 5 μm. (**c**–**f**) FRAP measurement of vesicle mobility within synaptic boutons. Representative images acquired immediately before photobleaching (pre-bleach) and immediately after bleaching at 0–60 s post-bleaching time points (**c**). Plot of fluorescence recovery (% of initial fluorescence) over time and fit with double-exponential curve (**d**). Plot of fast recovery rates calculated from fluorescence recovery curve (**e**) One-way ANOVA, **P=0.0011 (R406W), ***P=0.0001 (V337M), *P=0.0158 (P301L), *n*=24, 21, 22, 20, 25 (Control, WT, R406W, V337M, P301L) boutons (3–5 boutons per animal). Plot of slow recovery rates (**f**). One-way ANOVA, *P=0.0001 (R406W, V337M), *P=0.0250 (P301L), *n*=24, 21, 22, 20, 25 (Control, WT, R406W, V337M, P301L) boutons (3–5 boutons per animal). Scale bar, 5 μm. Data present mean±s.e.m. (**g**,**h**) Proposed model of Tau clustering synaptic vesicles to F-actin to restrict reserve pool vesicle mobilization and release.

While our data reveal that the N-terminal domain of Tau binds vesicles, it was also previously described that the proline-rich and microtubule-binding domains of Tau promote actin polymerization^48^,^49^. We therefore hypothesized that vesicle-binding and simultaneous F-actin formation by Tau may restrict synaptic vesicle mobility^50^. Accordingly, we find that the presence of Tau increases presynaptic F-actin levels using the genetic probe LifeAct-GFP (Fig. 3a,b). We then performed fluorescence recovery after photobleaching (FRAP) experiments to measure the mobility of synaptic vesicles labelled by Synaptotagmin-GFP within presynaptic boutons. Fluorescence recovery, and thus vesicle mobility, is significantly slower in animals expressing pathogenic mutant Tau compared to controls or animals expressing wild type Tau (Fig. 3c–f). The lower vesicle mobility defect in animals expressing mutant Tau is rescued when we incubate the preparations with the actin depolymerizing drug Latrunculin A to lower presynaptic F-actin levels (Supplementary Figs 6 and 7). These data confirm that increased presynaptic F-actin contributes to the impaired vesicle mobilization. We therefore propose a model in which pathogenic Tau behaves as a vesicle clustering molecule by interacting with vesicles and cross-linking them to F-actin to restrict vesicle mobility and release (Fig. 3g,h).

While pathogenic mutations in Tau lower its microtubule binding affinity, it is well-established that hyperphosphorylation of Tau in AD and related Tauopathies is the most common cause for impairments in microtubule binding^3^. We thus asked whether hyperphosphorylation of Tau affects synaptic vesicle dynamics in a manner similar to the pathogenic Tau mutants. We used D42-Gal4 to express UAS-Tau^E14^ in which 14 putative phospho-Serine/Threonine sites are mutated to glutamate^51^. Similar to the pathogenic mutants of Tau, we find phospho-mimetic Tau^E14^ to localize to presynaptic boutons of fly NMJs (Supplementary Fig. 8a,b). The presence of this Tau^E14^ at presynaptic terminals does not affect basal neurotransmitter release characteristics (Supplementary Fig. 8c,d), but it reduced synaptic transmission during a 10 Hz stimulation train (Supplementary Fig. 8e). Also similar to our observations with expression of pathogenic mutant Tau, FRAP experiments of synaptotagmin-GFP in animals that express Tau^E14^ indicates a lower synaptic vesicle mobility (Supplementary Fig. 8f,h). These data are in line with our model that increased levels of Tau at presynaptic terminals causes synaptic vesicle clustering defects. The defects we observe with Tau^E14^ are not because the phosphorylation of Tau affects synaptic vesicle binding. Indeed, the ability of *in vitro* phosphorylated Tau (by GSK3-β and CDK5) to bind synaptic vesicles in co-sedimentation assays is indistinguishable from that of non-phosphorylated Tau (Supplementary Fig. 9). Altogether these data suggest that hyperphosphorylated Tau can bind synaptic vesicles and can impair vesicle mobilization in a manner similar to pathogenic mutant Tau.

Given that the *Drosophila* models used in the current study ectopically express mutant Tau under a wild type genetic background, we questioned whether competition between mutant Tau and endogenous Tau could contribute to the detected phenotypes of vesicle mobility. To address this question, we analysed *tau*^*−/−*^ flies to assess the possible effects of endogenous Tau on vesicle mobilization. Tau knockout flies are viable and fertile, and they do not show defects in basal synaptic transmission at NMJs (Supplementary Fig. 10a–f), nor do they exhibit apparent defects in synaptic transmission during a high frequency (10 Hz) stimulation paradigm (Supplementary Fig. 10g). Moreover, FRAP experiments at the NMJs did not detect a vesicle mobility phenotype in *tau*^*−/−*^ flies (Supplementary Fig. 10h). Altogether, loss-of-function of endogenous Tau does not cause apparent vesicle mobilization phenotypes. Therefore, the impairments of vesicle mobility observed in mutant Tau-expressing animals does not appear to rely on competition with endogenous Tau, and our data support a gain-of-toxic function mechanism of Tau mislocalized to synaptic terminals.

### Inhibiting Tau-vesicle binding rescues presynaptic deficits

To test our model that Tau binds and clusters vesicles through its N-terminal domain, we generated transgenic flies that express N-terminally truncated pathogenic mutant Tau (ΔN_R406W, ΔN_V337M and ΔN_P301L) and assessed vesicle mobility and presynaptic function. These N-terminal truncation mutants all localize to presynaptic boutons (Supplementary Fig. 11a,b) and increase presynaptic F-actin levels (Supplementary Fig. 11e) to similar extents as their full-length counterparts. However, ΔN-pathogenic Tau mutants show a more diffuse localization, distinct from the CSP-labelled ‘doughnut-like' synaptic vesicle localization pattern (Supplementary Fig. 11a and c). These data corroborate the inability of ΔN-Tau to bind vesicles. We confirmed this observation using the *shi*^*ts1*^ vesicle depletion assay (see above and Fig. 1d). In stimulated *shi*^*ts1*^ mutants, CSP relocalizes to the plasma membrane but the ΔN-pathogenic Tau mutants remain diffusely present in the boutons (Supplementary Fig. 11d). Altogether, these data indicate reduced association of ΔN-Tau with synaptic vesicles *in vivo*.

**Figure 4:**
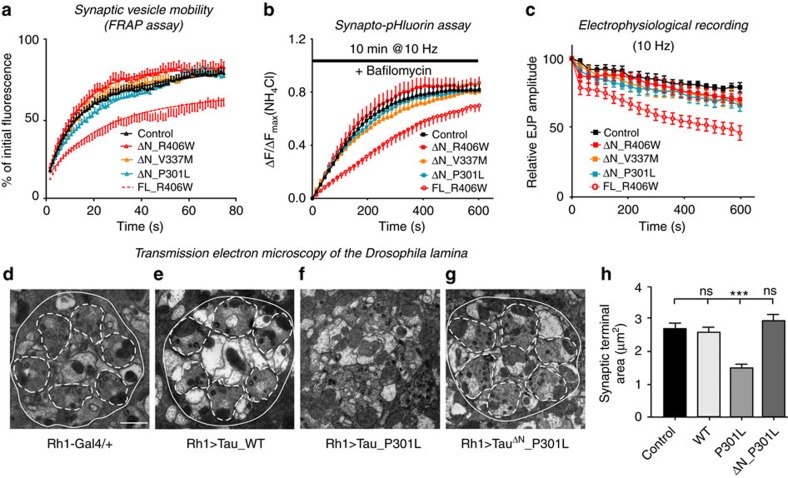
Interfering with Tau N-terminal-dependent vesicle-binding reverts Tau-induced presynaptic deficits in fly neurons. *Drosophila* larvae used in (**a**–**c**) express UAS-Tau^ΔN^ (R406W, V337M or P301L) under the D42-Gal4 motor neuron driver. (**a**) FRAP measurements of vesicle mobility within synaptic boutons. Fluoresence recovery (% of initial fluorescence) was plotted over time and fit with double-exponential curves. *n*=22 (R406W), 20 (ΔN_R406W, ΔN_V337M), 24 (ΔN_P301L) or 25 (Control) boutons (3–5 boutons per animal). (**b**) Synapto-pHluorin responses to stimulation at 10 Hz with the presence of bafilomycin. Fluorescence change ΔF at ratio to maximal Δ*F* (NH_4_Cl dequenching) was plotted over time during the stimulation. Two-way ANOVA, *n*=7 (R406W, ΔN_R406W, ΔN_V337M, ΔN_P301L),9 (Control) NMJs (animals). (**c**) Electrophysiological recordings of EJP amplitudes during stimulation at 10 Hz. Two-way ANOVA, *n*=7 (R406W, ΔN_R406W), 8 (ΔN_V337M, ΔN_P301L), or 9 (Control) NMJs (animals). (**d**–**h**) Transmission electron microscopy (TEM) of lamina sections of 9-day-old flies expressing UAS-Tau (WT, P301L or ΔN_P301L) under the late-onset retinal driver Rhodopsin1-Gal4 (Rh1). In control Rh1-Gal4/+ animals (**d**), six photoreceptor synaptic terminals (outlined in dashed white lines) are converged in a ‘cartridge' (outlined in white line). Disrupted synaptic terminal organization and morphology are detected in Rh1>Tau_P301L (**f**) flies, whereas no obvious abnormalities at the synaptic terminals of Rh1>Tau_WT (**e**) and Rh1>Tau^ΔN^_P301L (**g**) flies. Scale bar, 1 μm. (**h**) Quantification of the synaptic terminal area based on the electromicrographs. One-way ANOVA, ***P=0.0001. *n*=5 animals per genotype. Data present mean±s.e.m.

To assess vesicle mobility we used the Synaptotagmin-GFP FRAP assay. In contrast to synapses expressing full length pathogenic Tau, synapses expressing ΔN-pathogenic Tau mutants show Synaptotagmin-GFP fluorescence recovery rates that are similar to those measured in controls (Fig. 4a). Furthermore, animals expressing N-terminally truncated pathogenic Tau mutants also do not show defects in the rate of synaptic vesicle mobilization and in the size of the active synaptic vesicle pool as assessed by SpH in the presence of bafilomycin (Fig. 4b). Finally, expression of N-terminally truncated Tau mutants does not cause defects in maintaining neurotransmitter release during 10 Hz stimulation (Fig. 4c). Thus, the N-terminal domain of Tau mediates synaptic vesicle mobilization and the presynaptic defects upon expression of pathogenic Tau *in vivo.* Furthermore, our data also indicate that increased F-actin levels alone are not sufficient to cluster vesicles and that Tau binding to vesicles is needed as well.

We next assessed Tau-mediated neurodegeneration in the fly brain. Previous work reported vacuolar degeneration in brain sections of Tau transgenic flies^38^. However, our lower Tau expressing lines did not show this defect (Supplementary Fig. 12). Given the synaptic defects we observed in our study we resorted to electron microscopy to reveal presynaptic integrity. Photoreceptors in the fly brain lamina form clean topographic maps, with six presynaptic photoreceptor terminals organizing into one cartridge. In controls and animals expressing wild type Tau, electron micrographs reveal regular shaped cartridges (Fig. 4d,e). In contrast, the organization and morphology of presynaptic terminals are disrupted in animals expressing P301L mutant Tau (Fig. 4f,h). This presynaptic degeneration requires the N-terminal projection of Tau because cartridge morphology and presynaptic integrity is normal in ΔN_P301L Tau expressing animals (Fig. 4g,h). The data suggest that interfering with vesicle binding prevents mutant Tau-induced presynaptic degeneration in flies.

**Figure 5:**
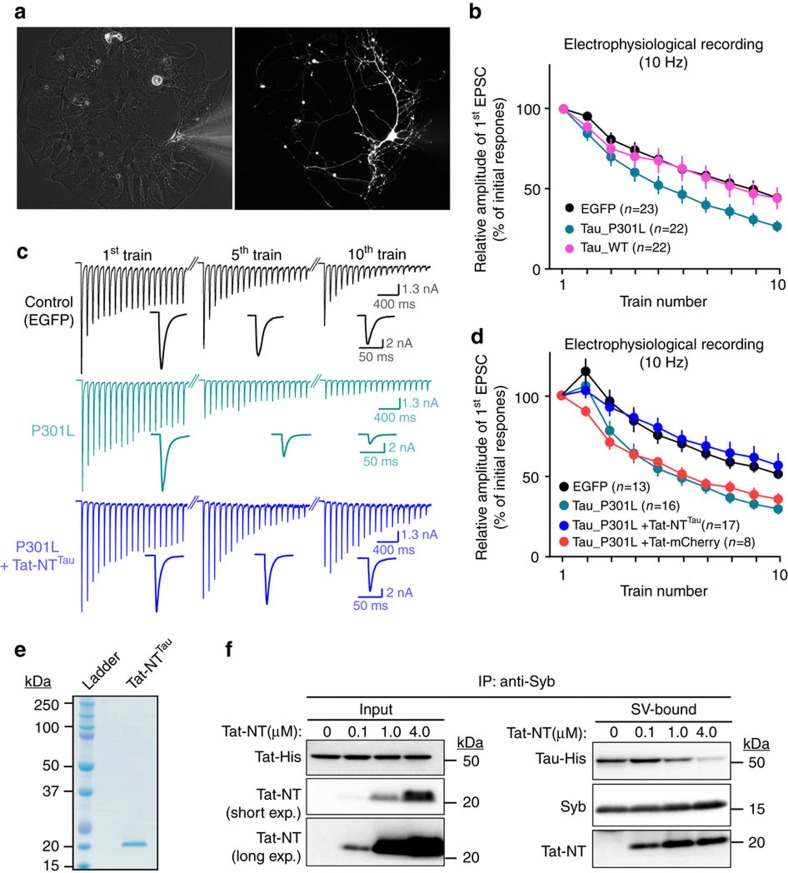
Interfering with Tau N-terminal-dependent vesicle-binding rescues Tau-induced presynaptic deficits in cultured rat hippocampal neurons. Autaptic rat hippocampal neuronal cultures were transduced with AAV viral vectors expressing GFP or Tau variants. (**a**) Autaptic neuronal culture transduced with AAV-EGFP is visualized by bright-field illumination (left) and GFP fluorescence (right). (**b**–**d**) Electrophysiological recordings using patch clamp in response to 10 consecutive high frequency stimulation trains (10 Hz for 10 s with 30 s interval). The representative traces are shown in (**c**) and the relative first EPSCs were plotted to train numbers (**b**,**d**). In (**d**), Tau^P301L^ expressing neurons were acutely treated with 5 μM Tat-NT^Tau^ or control Tat-mCherry peptides before patch clamp recordings. The experiments were independently repeated at least three times and the number of neurons recorded is indicated in the graph. Data present mean±s.e.m. (**e**) Colloidal coomassie staining of purified Tat-NT^Tau^ peptide; NT^Tau^ corresponds to the N-terminal domain (aa 1–113) of Tau. (**f**) Tat-NT^Tau^ peptide competes with full-length Tau for vesicle binding *in vitro*. Intact synaptic vesicles (SVs) were immobilized to Dynabeads using anti-Synaptobrevin 2 (Syb) antibodies. Following immobilization, SVs were incubated with recombinant full-length Tau (40 nM) with or without the presence of 0.1, 1.0 or 4.0 μM purified Tat-NT^Tau^ peptide. Immunoblots were probed for full-length Tau, Tat-NT^Tau^ and the synaptic vesicle marker Syb. Note that Tat-NT^Tau^ reduced the amount of full-length Tau bound to SVs in a dose-dependent manner.

We further confirmed our findings in rat hippocampal autaptic cultures^52^ (Fig. 5a) by transducing the neurons with AAV expressing either wild type (WT) or mutant Tau (P301L, R406W). As assessed by immunolabeling, both WT and mutant Tau are localized to neurites and similar to our observations in patient samples and *Drosophila*, mutant Tau (P301L, R406W) is more often present in synapsin-labelled presynaptic puncta (Supplementary Fig. 13). Also consistent with our observations at *Drosophila* neuromuscular junctions (Supplementary Fig. 4), whole cell voltage clamp recordings of rat neurons expressing wild type Tau or mutant Tau does not show significant differences in basal neurotransmitter release parameters (Supplementary Figs 14 and 15a–d). We then measured neurotransmitter release in response to 10 consecutive high-frequency stimulation trains assessing the ability of vesicle pools to mobilize in order to sustain release (10 Hz for 10 s with 30 s intervals). Neuronal cultures transduced with AAV-Tau^WT^ maintain release efficacy similar to cells transduced with control AAV expressing GFP (Fig. 5b,c). In contrast, neurons expressing pathogenic Tau (P301L or R406W) are unable to maintain release (Fig. 5b,c and Supplementary Fig. 15e,f), in agreement with our observations in *Drosophila*.

Next, we designed and purified a peptide corresponding to the human Tau N-terminal domain (NT^Tau^, aa_1–112_) fused to a minimal HIV-derived cell-penetrating Tat peptide (Fig. 5e), which is able to compete for synaptic vesicle binding with full-length Tau *in vitro* (Fig. 5f). Acute treatment of neurons with the Tat-NT^Tau^ peptide does not affect their basal release characteristics (Supplementary Fig. 14). Similar to our results obtained in *Drosophila*, we find increased F-actin levels in Tau_P301L-expressing neurons compared to controls (Supplementary Fig. 16), which is unaffected by treatment with the Tat-NT^Tau^ peptide. Hence, in agreement with the fly data, interfering with the ability of the N-terminus of Tau to bind synaptic vesicles does not affect Tau-induced actin polymerization at presynaptic terminals.

We next assessed the effect of Tat-NT^Tau^ treatment on neurotransmitter release during 10 Hz stimulation trains, and we find that Tat-NT^Tau^ significantly restores the defect in neurotransmitter release in neurons expressing Tau_P301L, while adding a control Tat-mCherry peptide has no effect (Fig. 5d). In conclusion, both fly and rat neurons display comparable presynaptic defects upon expression of pathogenic Tau, but not expression of wild type Tau, and our data indicate that blocking the ability of the Tau N-terminal domain to interact with synaptic vesicles rescues the presynaptic defects induced by pathogenic Tau.

## Discussion

Our work demonstrates that pathogenic mutant Tau alters presynaptic properties, complementing previous findings that expression of mutant Tau affects postsynaptic functions^18^,^21^. Both wild type and pathogenic mutant Tau can bind synaptic vesicles to a similar extent *in vitro*, but expression of the mutant form leads to increased localization of Tau to presynaptic terminals. In addition to pathogenic mutations, abnormal phosphorylation of Tau in AD also results in the dissociation of Tau from microtubules. At fly NMJs, phospho-mimetic Tau^E14^ behaves similar to pathogenic mutant Tau, as it mislocalizes to presynaptic terminals and impairs vesicle mobilization. Our data also show that pathologically phosphorylated Tau binds to synaptic vesicles *in vitro*. This again supports the notion that phosphorylation or clinical mutations drive Tau to the synapse where it comes in contact with vesicles, but does not directly affect the binding affinity of Tau to vesicles; this also makes sense in light of the fact that Tau phosphorylation sites (mostly within the proline-rich and C-terminal domains) and sites of disease-causing mutations in Tau (clustering around the microtubule binding domain) do not overlap with the vesicle binding domain (the N-terminal projection) that we identified here. Therefore, we propose that this mechanism may be common to various Tauopathies associated with abnormal phosphorylation or mutations of Tau.

We propose a model in which when Tau is present presynaptically, it binds to vesicles with its N-terminal domain and polymerizes presynaptic actin using its proline-rich and microtubule-binding domains. Tau thereby crosslinks synaptic vesicles by binding vesicles and actin, and slows their mobilization, lowering synaptic transmission during intense stimulation. We suggest a multi-step mode of action in which disease conditions lead to the dissociation of Tau from microtubules, followed by an early soluble phase where the protein induces pathological synaptic dysfunction presynaptically as we show here. Indeed, recent studies in Tau transgenic *Drosophila* and mice both detected early synaptic dysfunction preceding pathological aggregation and neurodegeneration^23^,^53^, and we propose here that the pathway identified in the current work may underlie early presynaptic dysfunction, triggering a cascade of events ultimately leading to synaptic and neuronal loss. We now show that Tau N-terminal-dependent vesicle binding is a key mechanism that induces presynaptic defects in the absence of tangle formation. Our work also suggests that interfering with Tau-N-terminal dependent vesicle-binding could be exploited therapeutically to prevent these aspects of presynaptic pathology.

The discovery of a function for the N-terminal projection dissociates the vesicle binding and actin polymerization role of pathogenic Tau at synapses. The N-terminal domain of Tau is dispensable for actin polymerization because ΔN-pathogenic Tau mutants localize to presynaptic terminals and still support the formation of excess F-actin at synapses. These results also indicate that Tau-induced actin polymerization at synapses alone is not sufficient to measurably hinder vesicle mobilization; rather, the direct binding of Tau to synaptic vesicles is also needed. Notably, Tau also dimerizes and multimerizes ahead of tangle formation and while not studied further here, this activity could potentially also add to the ability of Tau to impede with vesicle mobility. Interestingly, a similar pathogenic function, the direct cross-linking of vesicles by multimerization, has been suggested for α-synuclein^54^,^55^, a protein that aggregates in Lewy body disease and Parkinson's disease, suggesting that Tau and α-synuclein may harbour overlapping effects on presynaptic function.

Our findings suggest that Tau employs a similar mode of vesicle immobilization as Synapsins, which also bind vesicles and F-actin to keep vesicles in a reserve pool^50^. Consistent with this function, we observed a defect to maintain normal levels of neurotransmitter release in fly and rat neurons expressing pathogenic Tau. This neurotransmitter release defect is similar to that seen upon overexpression of Synapsin, as in different model synapses, expression of Synapsin has been shown to result in a decline of neurotransmission in response to strong stimulation trains^56^,^57^. There are previous studies showing reduced amplitudes of mEPSCs in primary neurons expressing mutant Tau at DIV 22–30 (refs 21, 58). In our study, at much earlier time points (DIV 11–13), we did not detect such a change in mEPSCs. The discrepancy is likely due to the postsynaptic accumulation of mutant Tau in older neuronal cultures, in which dendritic spines are more mature and relatively stable^59^,^60^. Conversely, the dendritic protrusions in younger neurons are rather transient and highly dynamic^59^,^60^. Thus we surmise our electrophysiological recordings at DIV 11–13 mainly reflect the effects of Tau on presynaptic vesicle release, where we did not detect a deficit at basal level. There are also electrophysiological studies performed in Tauopathy mouse models, but mostly at a stage where substantial synapse and neuron loss had already occurred and these studies also often focused on the post-synaptic role of Tau^18^,^61^,^62^,^63^. Consistent with our observations that expression of Tau lowers neurotransmitter release efficiency, more recent studies in mice overexpressing Tau_P301L indicated early stage defects and show a loss of synaptic input before synapse loss in intact neocortical pyramidal cells as well as alterations in the probability of neurotransmitter release in entorhinal cortex^19^,^23^. While a connection between vesicle mobilization defects and neuronal death awaits further investigation, it is conceivable that a defect to maintain normal release contributes to the early synaptic defects seen in neurodegenerative disease. The strength of the defects we observed are also subtle enough to explain the occurrence of disease symptoms only later in life. In addition, in humans, long lasting high-frequency activity has been observed during cognitive tasks^64^,^65^,^66^, suggesting that Tau-induced vesicle mobilization defects and the inability to maintain normal levels of neurotransmitter release could potentially contribute to cognitive decline in dementia.

A number of recent studies show that Tau N-terminal fragments, including a 17-kDa (Tau44–230) fragment and a 35-kDa (Tau1–391) fragment, are elevated in the brains of Tauopathy patients^58^,^67^. These two fragments are generated by Calpain and Caspase-2 cleavage of Tau, and they are shown to be neurotoxic in cellular and animal models^58^,^67^. The 35-kDa fragment acts similar to soluble pathogenic Tau, as it binds less efficiently to microtubules and missorts to dendritic spines to impair synaptic function^58^. It remains to be assessed, but it is likely, that the 35-kDa fragment also missorts to presynaptic terminals, where it can bind to synaptic vesicles and simultaneously promote actin polymerization, as it contains the required domains for both vesicle- and actin-binding. Whether the 17-kDa fragment causes synaptic toxicity remains unknown, but it has been shown to inhibit aggregation of full-length Tau^67^. Thus the presence of the 17-kDa fragment may potentially lead to an increase of soluble Tau under disease conditions, and subsequently more Tau missorting to the synapse. Our work shows that increased soluble Tau at presynaptic terminals impairs vesicle dynamics, which could be a possible mechanism that contributes to the toxicity of these N-terminal fragments. Future work should assess these possibilities.

Our work indicates that targeting the N-terminal domain of Tau is sufficient to alleviate the presynaptic defects induced by the expression of pathogenic Tau. Indeed, expression of mutant Tau proteins lacking the N-terminal projection does not elicit presynaptic defects anymore. We therefore reasoned that tools that compete with Tau for vesicle binding would be beneficial. Treating rat neurons that express Tau_P301L with a membrane permeable N-terminal Tau domain (Tat-NT^Tau^ peptide), shown to compete for synaptic vesicle binding, rescues the pathological Tau-induced inability to maintain neurotransmitter release. Our work provides proof-of principle that it is possible to target the ability of Tau to interact with vesicles to rescue presynaptic defects elicited by the excessive presynaptic localization of pathogenic Tau. These findings also pave the way for the development of more specific tools with higher affinity and specificity. In relation to this, while short-term exposure to the current N-terminal peptide itself did not elicit obvious neuronal toxicity, it will also be essential to assess if longer exposure of tools that inhibit Tau vesicle binding affect aspects of presynaptic or other neuronal function for their therapeutic potential. Nonetheless, our work reveals a new aspect of Tau biology and suggests there is a window to tackle the presynaptic defects induced by pathological Tau.

## Methods

### Drosophila genetics

All *Drosophila melanogaster* stocks and experimental crosses were kept on standard corn meal and molasses food. Stocks were kept at room temperature. Experimental crosses involving UAS/GAL4 bipartite expression were kept at 25 °C. All UAS-human Tau (0N4R) flies used in this work were generated by insertion of transgene into a same genomic locus (68A4) and were expressed under the motor neuron-specific D42-Gal4 driver. The UAS-Tau_WT and UAS-Tau_R406W fly lines were obtained as gifts from lab of Dr. Guy Tear. The UAS-Tau_V337M and UAS-Tau_P301L fly lines were generated in this current work. Additional stocks used in this study include UAS-Synapto-pHluorin, UAS-LifeAct-GFP (BSC 57326) and UAS-synaptotagmin-eGFP (BSC 6925) from Bloomington Stock Center. UAS-hTau_WT(H), UAS-hTau_R406W (H) and UAS-hTau_E14 (III) fly lines were obtained as gifts from lab of Dr. Mel Feany. Tau knockout fly line was generated by genome editing^68^.

### Synapto-pHluorin imaging at fly NMJs

Third-instar *Drosophila* larvae were dissected in Ca^2+^-free HL3 saline (110 mM NaCl, 5 mM KCl, 10 mM NaHCO_3_, 5 mM HEPES, 30 mM sucrose, 5 mM trehalose, 10 mM MgCl_2_, pH 7.2) and then incubated in HL3 supplemented with 1 mM calcium and 500 μM 1-Naphthylacetyl spermine trihydrochloride (Sigma) to prevent muscle contractions^69^. Motor nerves were stimulated at × 2 threshold using an Axoclamp 900A amplifier, and images were captured though a water-dipping objective (× 40, 0.8N.A.) using a cooled CCD camera (Andor Clara DR-328G-CO1-SIL) mounted on a Nikon ECLIPSE FN1 microscope. Images were analysed using NIS-Elements AR3.2 software. To assess vesicle exocytosis and endocytosis, motor nerves were stimulated at 10 Hz for 5 s and images were captured at 900 ms intervals before, during and after stimulation. The fluorescence changes (ΔF spH) reflect the intensities of boutonic fluorescence at corresponding time points subtracting the basal fluorescence intensities before stimulation. To assess endocytosis, ΔF was normalized by ratio to peak ΔF and the fluorescence decays were fitted in two-phase curves for fast and slow time constants to assess the rates of endocytosis. To assess vesicle release during sustained stimulation, 0.5 μM bafilomycin (Merck Millipore) were added into the incubation bath of dissected larva. Motor nerves were stimulated at 10 Hz for 10 min and images were captured at 15 s intervals. After stimulation, 50 mM NH_4_Cl (pH7.4) was added to dequench spH fluorescence signals. The ratios of ΔF/ΔFmax (NH_4_Cl) were plotted to corresponding time points. In all experiments the NMJs at muscles 12 and 13 in segments A2–A4 were selected for imaging. Data are expressed as average±s.e.m.

### FM1-43 dye labelling

Third-instar larvae were dissected in Ca^2+^-free HL3 saline on Sylgard-coated plates. Motor nerves were stimulated at × 2 threshold using an Axoclamp 900A amplifier to load FM1-43 dye (4 μM; Molecular Probes) in HL3 solution supplemented with 1 mM Ca^2+^. FM dye loading of NMJs at muscle 12/13 in segments A2-A4 was imaged using a × 60 1.0N.A. water immersion lens on a Nikon fluorescent microscope. The microscope filter was set for FM1-43 emission and excitation.

### Electrophysiological recording at fly NMJs

Intracellular voltage recordings from third-instar larval muscle 12 in segment A2 or A3 were performed using ∼20 MΩ sharp electrodes and stimulation at × 2 threshold. EJPs and spontaneous miniature junctional potentials (mEJPs) were recorded with an Axoclamp 900A amplifier digitized using a Digi-data 1440A and stored using pClamp 10.2 software (Molecular Devices). For quantification of 10 Hz recordings, EJP amplitudes were binned per 30 s and averages are normalized to the amplitude measured in the first 15 stimuli.

### Fluorescence recovery after photo-bleaching assay

Experiments were performed on third-instar larval fillets dissected in HL3 saline on sylgard-coated plates. For FRAP analysis, all images were captured within one hour after dissection of the larval, using a Nikon A1R confocal laser microscope equipped with a × 60 1.0N.A. water immersion objective. The vesicle dynamics were recorded from type Ib boutons on NMJs at muscles 12 and 13, with 3–5 boutons from the same larvae used. Images were acquired at 1.12 μs per pixel with a pinhole of one airy unit and a resolution of 512 × 512. A region of interest (ROI) 24 × 30 pixels was selected on the digital image for photo-bleaching. Four baseline scans were performedusing 5% of full laser power. Immediately before the fifth scan, the laser power were increased to 95% of maximal and rapidly iterated the ROI for nine times. After the photo-bleaching, the laser was returned to 5% of maximal power to complete the remaining scans. Fluorescence intensity of the bleached region, the selected background region and the reference bouton were analysed using NIS-Elements AR3.2 software with the Time Series Analyzer plug-in. The recovery curves were fitted a double exponential curve, which was further used to to account for the two-phasefluorescence recovery of the initial faster recovery immediately after bleaching and the later plateau observed with respect to vesicle mobility. For FRAP experiments performed with Latrunculin A (LatA) treatment, *Drosophila* larvae were dissected in HL3 buffer then bathed in HL3 buffer with 10 μM LatA (sea sponge origin, Sigma L5163) for 5–7 min, followed by FRAP measurements in the same buffer containing Lat A. Live imaging was carried out in <30 min following onset of Lat A treatment.

### Vesicle depletion in shi^ts1^ mutant background

Third-instar larvae were dissected in Ca^2+^-free HL3 buffer on a sylgard dish at room temperature. The sylgard dish with the dissected preparation in Ca^2+^-free HL3 buffer was placed in a 34 °C oven, and the buffer was replaced by pre-warmed Ca^2+^-free HL3 buffer (34 °C). After allowing 3–5 min for temperature to equilibrate, the preparation was stimulated in pre-warmed high K^+^ (HL3 buffer supplemented with 60 mM KCl and 1.5 mM Ca^2+^) at 34 °C for 10 min. This stimulation was followed by 5 min incubation in pre-warmed Ca^2+^-free HL3 at 34 °C. The samples were then fixed in pre-warmed 4% PFA at 34 °C for 10–15 min and proceed with washing and immunofluorescence staining. Images were obtained with a Zeiss ELYRA super resolution microscope.

### Immunohistochemistry

Third-instar *Drosophila* larvae were dissected in fresh HL-3 solution and fixed in 3.7% formaldehyde for 20 min at room temperature. After washing with PBS, tissue was permeabilized with PBS containing 0.4% TritonX-100 (PBT) for 1 h, followed by blocking in 1% BSA for 1 h at room temperature. Immunostaining was performed with the following primary antibodies: DAKO anti-total tau rabbit pAb (DAKO, A0024, 1:1,000), anti-CSP2 mouse mAb (DSHB, 6D6, 1:1,000) and anti-GFP rabbit pAb (Invitrogen, A-11122, 1:1,000) for probing LifeAct-GFP. The secondary antibodies used were: goat anti-rabbit Alexa 488 (Invitrogen, 1:5,000) and goat anti-mouse Alexa 555 (Invitrogen, 1:5,000). Following antibody labelling, the preparations were washed in PBT and mounted in Vectashield (Vector Laboratories). Samples were visualized on a Nikon confocal microscope or a Zeiss ELYRA super resolution microscope.

### Immunoblotting

All protein samples were reduced in × 1 lithium dodecyl sulfate (LDS) sample buffer (Invitrogen) supplemented with 1% β-mercaptoethanol for 10 min at 70 °C. Proteins were separated on NuPAGE Novex 4–12% Bis-Tris polyacrylamide gels (Invitrogen) in MOPS buffer and then transferred to nitrocellulose membrane using the Trans-Blot Turbo transfer system (BioRad). For immunoblotting, membranes were blocked in Tris-buffered saline+0.05% Tween-20 (TBST) with 5% milk powder for 1 hour at room temperature before incubation with primary antibodies diluted in blocking buffer. The following primary antibodies were used in this study: DAKO against total Tau (DAKO, A0024, 1:1,000), anti-Synaptobrevin 2 (Synaptic Systems Clone 69.1, 1:1,000), anti-Synaptotagmin (DSHB, Asv48, 1:1,000), anti-Synapsin (Merck Millipore AB1543P, 1:1,000), anti-Synaptophysin (Synaptic Systems Clone 7.2, 1:1,000) and anti-Tubulin (DSHB, E7, 1:5000). After incubation with primary antibodies, HRP-conjugated species-specific secondary antibodies were added at a concentration of 1:10,000 for 1 hour at room temperature. Signal was detected using the Western Lightning Plus ECL kit (Perkin Elmer) and imaged on a Fuji-Film digital imaging system. Uncropped immunoblot images for all blots in this study can be found in Supplementary Fig. 17.

### Ultrapure synaptic vesicle purification from rat brains

Synaptic vesicles were purified using differential centrifugation and size exclusion chromatography^70^. The whole procedure was carried out on ice or at 4 °C to minimize proteolysis. Briefly, 20 rat brains weighing between 150 and 180 g werecollected, and thetissue was homogenized in a motor driven glass-teflon homogenizer at 900 r.p.m., using 240 ml ice-cold sucrose buffer (320 mM sucrose, 4 mM HEPES (pH 7.4, NaOH)) supplemented with 0.2 mM phenylmethylsulfonylfluoride (PMSF) and 1 mg ml^−1^ pepstatin A. The homogenate (H) was centrifuged for 10 min at 800 g Av to yield a pellet (P1). The supernatant was further centrifuged for 15 min at 12,000 g Av to generate the P2 pellet, which was washed twice with sucrose buffer, yielding a clean synaptosome pellet (P2′). The synaptosomal pellet was resuspended in 24 ml sucrose buffer and lysed by addition of 9 volumes distilled H_2_O, followed by homogenization. Five mM HEPES (pH 7.4, NaOH) was added, supplemented with 0.2 mM PMSF and 1 mg ml^−1^ pepstatin A. This lysate was then centrifuged for 20 min at 32,500 g Av giving a pellet (LP1) and supernatant (LS1). The supernatant was further centrifuged for 2 h at 230,000 gAv, yielding a crude synaptic vesicle pellet (LP2). This crude synaptic vesicle pellet was resuspended in 40 mM sucrose and further centrifuged for 4 h at 82,500 gAv on a continuous sucrose density gradient (50–800 mM sucrose). Vesicles were collected from the gradient and subjected to size-exclusion chromatography on controlled pore glass beads (300 nm diameter), equilibrated in 300 mM glycine, 5 mM HEPES (pH 7.4, KOH), to separate synaptic vesicles from residual myelin and larger membrane contaminants. Synaptic vesicles were pelleted by centrifugation for 2 h at 230,000 g Av and resuspended in HB150 buffer (150 mM KCl, 1 mM DTT, 25 mM HEPES (pH 7.4, KOH)) by homogenization. Aliquotes of the resuspended synaptic vesicles were snap frozen in liquid nitrogen. Electron microscopy (EM) and western blotting were applied to assess the purity of synaptic vesicles. For EM analysis, synaptic vesicles were absorbed to formvar-coated grids, fixed with 1% paraformaldehyde, quenched with 20 mM glycine and immunostained for synaptophysin (Clone G95). Protein A-gold was used for detection. After counterstaining with 1% uranylacetate, samples were imaged using a CM120 electron microscope, equipped with a TemCam 224A CCD camera. 95% plus of structures were immunopositive for synaptophysin and were 40–50 nm in diameter, which is the typical size for synaptic vesicles. For western blotting, the protein concentration of collected fractions was determined using a modified Lowry assay using BSA as a standard. Approximately 10 μg total protein was separated using a tricine based gel system, and then transferred to nitrocellulose membrane using standard semi-dry techniques. Membranes were blocked and incubated with primary antibodies (anti-synaptophysin, clone 7.2, Synaptic Systems; anti-NMDA-Receptor 1, clone M68, Synaptic Systems) overnight at 4 °C. HRP-conjugated secondary antibodies were then added for one hour at room temperature. Blots were developed using Western Lightning chemiluminescence reagents and images acquired using a Fuji-LAS reader. As expected, the purified synaptic vesicle fraction showed enrichment of synaptophysin and depletion of NMDA-R1.

### Synaptic vesicle sedimentation assay

To perform the co-sedimentation assay, 500 ng of freshly purified recombinant human Tau-His was incubated with an excess amount of ultrapure synaptic vesicles (equivalent to 20 μg of protein) prepared from rat brains in synaptic vesicle binding buffer (4 mM HEPES pH 7.4, 5 mM Tris–HCl pH 7.4, 220 mM glycine, 0.1% BSA, protease inhibitors) at 4 °C for 2 h in a volume of 100 μl. After 2 h of incubation, vesicles were pelleted by ultra-centrifugation at 400,000 *g* for 30 min. Pellets were then re-suspended and reduced in LDS sample buffer. The presence of recombinant human Tau-His in the synaptic vesicle pellet was assessed by immunoblotting using anti-His antibodies.

### Transmission electron microscopy

Adult fly heads were dissected and immediately fixed in 4% paraformaldehyde and 2% glutaraldehyde in 0.1 M Na-Cacodylate buffer (pH 7.4) for 2 h at room temperature. Samples were further fixed at 4 °C overnight, and then washed with 0.1 M Na-Cacodylate, pH 7.4, and subsequently osmicated with 2% osmium (OsO_4_/Na-Cacodylate buffer). After dehydration in an ascending series of ethanol solutions and staining in 4% aqueous uranyl acetate solution the specimen were embedded in Agar 100 (Laborimpex; Agar Scientific). Ultrathin sections (70 nm) of the retina and the lamina were collected on grids (Laborimpex; Agar Scientific) coated with Butvar and imaged on a JEM 1400 transmission electron microscope (JEOL) at 80 kV with a bottom mounted camera (Quemasa; 11 megapixels; Olympus) running iTEM 5.2 software (Olympus).

### Rat neuronal cultures

Wistar rat embryos (Janvier Labs) of embryonic day 18 (E18) were dissected in hanks buffered salts solution (HBSS; Sigma) buffered with 7 mM HEPES, and the hippocampi were collected in the dissection buffer. After removal of the meninges, hippocampi were minced and incubated in 0.25% trypsine in HBSS for 15–20 min at 37 °C. After washing the neurons were triturated, counted and plated in Neurobasal (E18 neurons) medium (Invitrogen, Carlsbad, USA) supplemented with 2% B-27 (Invitrogen), 1.8% HEPES, 1% glutamax (Invitrogen), 1% Pen/Strep (Invitrogen) and 0.2% β-mercaptoethanol. Neurons were plated at 2,500 cells cm^−2^ on mouse glia microislands. Glial islands were prepared by first coating glass coverslips with 0.15% agarose. After drying and ultraviolet sterilization, custom-made rubber stamps were used to print dots (islands, diameter 200–250 μm) using a substrate mixture containing 0.25 mg ml^−1^ rat tail collagen and 0.4 mg ml^−1^ poly-D-lysine dissolved in 17 mM acetic acid; glial cells were plated at 4,800 cells cm^−2^. Neurons were transduced (DIV 5) with bicistronic AAV virus co-expressing Tau and GFP (to indicate transduced neurons), or transduced with control AAV virus expressing GFP alone. For competition experiments, transduced neurons expressing Tau_P301L were pretreated with 5 μM Tat-NT^Tau^ or Tat-mCherry peptide for four hours before recording.

### Electrophysiological recordings of rat neurons

Cultured hippocampal neurons from Wistar rat embryos were recorded on DIV 11-13. The intracellular pipette solution contained (in mM): 136 KCl, 18 HEPES, 4 Na-ATP, 4.6 MgCl_2_, 4 K_2_-ATP, 15 Creatine Phosphate, 1 EGTA and 50 U/ml Phospocreatine Kinase (300 mOsm, pH 7.30). The extracellular solution used during recordings contained the following components (in mM): 140 NaCl, 2.4 KCl, 4 CaCl_2_, 4 MgCl_2_, 10 HEPES, 10 Glucose (300 mOsm, pH 7.30). Only islands with singular neurons were recorded (whole-cell voltage clamped at −70 mV) using a double EPC-10 amplifier (HEKA Elektronik, Lambrecht/Pfalz, Germany) under control of Patchmaster v2 × 32 software (HEKA Elektronik). Currents were recorded at 20 Hz and low-pass filtered at 3 kHz when stored. Pipettes were pulled using a Sutter P-1000 and resistance ranged from 3 to 5 MΩ. The series resistance was compensated to 75–85%. Cells with series resistances above 15 MΩ were excluded for analysis. All recordings were performed at room temperature. Spontaneous glutamatergic release (mEPSCs) was recorded at −70 mV. Evoked release was induced using brief depolarizatingof the cell soma (from −70 to 0 mV for 1 ms) to initiate action potential dependent glutamatergic release (eEPSCs).

### Adeno-associated viral vectors

The complementary DNA (cDNA) of HA tagged wild type or mutant human Tau (0N4R) was subcloned into a bicistronic adeno-associated viral serotype 6 (AAV6) vector containing eGFP cDNA. The co-expression of eGFP and HA-Tau was driven by a human synapsin-1 promoter (Genscript, Piscataway, NJ, USA). Recombinant AAV vectors were propagated in HEK293 cells, purified by iodixanol step gradient ultracentrifugation and heparin affinity FPLC, followed by extensive dialysis against PBS. Genome copies were determined by quantitative real time PCR and purity>99% by SDS gel electrophoresis and silver staining.

### Array tomography of human brain tissue

Human brain samples from superior temporal gyrus were provided by collaborating neuropathologists Prof. Colin Smith (University of Edinburgh Sudden Death Brain Bank) and Prof Matthew Frosch (Massachusetts ADRC brain bank). Use of human tissue followed national and institutional ethics guidelines, and was approved by the Edinburgh Brain Bank Ethics Committee and the Academic and Clinical Central Office for Research and Development medical research ethics committee. Brain tissue was collected at autopsy, small tissue blocks of approximately 1 × 1 × 5 mm^3^ containing all six cortical layers from the pia to white matter were cut with a razor blade, and samples were fixed in 4% paraformaldehyde with 2.5% sucrose for 2–3 h. Tissue was then dehydrated and embedded in LR white resin. Embedded blocs were cut with an ultramicrotome (Leica Ultracut) into ribbons of serial ultrathin (70 nm) sections, which were mounted on gelatin subbed coverslips (Fisher #1.5). Sections were blocked in 0.05% Tween and 0.1% BSA in TBS for 30 min followed by overnight incubation with primary antibodies diluted in blocking buffer: Synapsin I (1:100, rabbit, Millipore AB1543) and AT8 (1:50, mouse, Thermo Scientific MN1020). The next day, sections were stained with for 30 min in the following secondary antibody solutions diluted 1:50 in blocking buffer: Alexa594-conjugated donkey anti rabbit antibodies (Invitrogen A21207) and Alexa488-conjugated donkey anti mouse antibodies (Invitrogen A21202). DAPI (0.005 mg ml^−1^) was included in the secondary antibody solution to stain nuclei. Images were acquired on a Zeiss axio Imager Z2 epifluorescence scope. A tilescan image was acquired with a × 10 objective of DAPI staining on the entire ribbon. High resolution images were acquired in the same location on every section on the ribbon using a × 63 oil 1.4 NA objective. Same exposure times were used to capture all images (chosen on an AD case with tau pathology).

### Immunoblotting of *Drosophila* and human brain samples

To compare the expression of human Tau protein in fly heads, UAS-human Tau fly lines from the current work (UAS-Tau^WT^_68A, UAS-Tau^R406W^_68A, UAS-Tau^V337M^_68A and UAS-Tau^P301L^_68A) and from the previous work (UAS-Tau^WT^_H and UAS-Tau^R406W^_H)^35^,^36^,^37^,^38^ were crossed to pan-neuronal driver Elav-Gal4 and maintained at 25 °C. The offspring from were collected on the day of eclosion and the fly heads were homogenized in RIPA buffer (10 mM Tris-Cl pH 8.0, 1 mM EDTA, 0.5 mM EGTA, 1% Triton X-100, 1% SDS, 140 mM NaCl, complete protease inhibitor). Lysates were incubated on ice for 15 min and centrifuged at 14,000 r.p.m. for 15 min at 4 °C. Protein concentration was determined using the BCA protein assay (Thermo Scientific). Samples were separated by SDS–polyacrylamide gel electrophoresis (SDS–PAGE) and analysed by immunoblotting (described above) for total Tau (DAKO, A0024, 1:1,000) and Tubulin (DSHB, E7, 1:5,000). The expression of human Tau protein in fly heads is further compared to that in human brains. Control human autopsy brain tissue from entorhinal cortex region was obtained from London Neurodegenerative Disease Brain Bank. Human brain lysates were prepared by homogenization in 2% SDS by Fastprep Machine on a program of 45 s- 6000 shakes per s and cleared by centrifugation at 14,000 r.p.m. for 15 min at 4 °C. Protein concentration was determined using BCA protein assay (Thermo Scientific). Equal amount of total proteins (15 μg) from both fly brains and human brains were separated by SDS–PAGE and analysed by immunoblotting (above) for total Tau (DAKO, A0024, 1:1,000) and Tubulin (DSHB, E7, 1:5,000). The relative Tau expression levels were quantified based on the intensities of protein bands from the immunoblots as the data represents the Tau protein levels from equal amounts of total protein from fly heads and human brains used for loading on SDS–PAGE.

### Isolation of synaptic vesicles from mouse brain

All animal experiments were performed with ethical permission from and under the guidelines of the KU Leuven animal ethics committee. To prepare synaptic vesicles, brains of six-week old wild type mice were isolated, pooled and homogenized in ice-cold homogenization buffer (320 mM sucrose, 4 mM HEPES pH 7.4, Complete protease inhibitors; 2 ml buffer per brain) with a Potter-Elvehjem Teflon glass homogenizer by 10 strokes at 600 r.p.m. Homogenates were centrifuged at 800 *g* for 10 min to remove nucleus and intact cells, and the supernatants were recovered and further centrifuged at 10,000 *g* for 15 min. The pellet was washed once with homogenization buffer and collected again by centrifugation at 10,000 *g* for 15 min. The washed pellet fraction (P2′) was then resuspended in homogenization buffer (100 μl per each hemisphere), and mixed with 9 volumes of ice-cold water (supplemented with 5 mM HEPES-KOH, pH 7.4 and protease inhibitors) to induce osmotic shock with mixing during rotation for 30 min 4 °C and then subjected to centrifugation at 25,000 *g* for 20 min. The pellet fraction (LP1) was discarded, and the supernatant (LS1) was further centrifuged at 200,000 *g* for 2 h to collect the crude synaptic vesicle fraction (LP2), which was resuspended in 5 mM HEPES pH 7.4, 300 mM glycine. All manipulation and centrifugation procedures were performed at 4 °C, and buffers were supplemented with fresh Complete protease inhibitor cocktail. To check vesicle enrichment, fractions collected during the vesicle preparation were lysed in RIPA buffer (Sigma) with protease inhibitors. Protein concentrations were measured and equal amounts of protein were reduced in LDS sample buffer and probed for presynaptic and postsynaptic membrane markers by immunoblotting.

### Purification of recombinant human Tau

The cDNAs encoding full-length or domain-truncated human Tau (0N4R isoform) were cloned into the *BamHI* and *EcoRI* restriction sites of pGEX-6P-1 (GE Healthcare) using a reverse primer encoding a 8x-His tag. The resulting fusion proteins contained an N-terminal GST tag followed by a PreScission Protease cleavage site, Tau and a C-terminal His tag. The plasmid was transformed into Rosetta bacteria (Merck Millipore) for recombinant expression. For expression, bacteria were inoculated in LB medium containing ampicillin and chloramphenicol and grown overnight at 37 °C. The next day, bacteria were diluted to an OD_600_ of 0.2, and let grow at 37 °C until the culture reached an OD_600_ of 1.0, at which point IPTG (Thermo Scientific) was added to a final concentration of 0.4 mM to induce recombinant protein expression. Bacteria were incubated for 2 h at 37 °C following induction, then were immediately pelleted and stored at −80 °C until lysis. For cell lysis, frozen bacterial cell pellets were resuspended in bacterial lysis buffer (PBS supplemented with 10% glycerol, 1% Triton-X-100, 1 mM PMSF, Complete protease inhibitors (Roche), lysozyme and Benzonase (Sigma) and incubated at 4 °C for 30 min with rotation, followed by centrifugation at 16,000 *g* for 20 min. Cleared cell lysates were loaded onto pre-washed Glutathione Sepharose 4B (GE Healthcare) and incubated for 2 hours at 4 °C with gentle rotation. After the incubation, the resin was washed twice with PBS supplemented with 250 mM NaCl, and then twice with PreScission Protease cleavage buffer (20 mM Tris–HCl pH 7.0, 50 mM NaCl, 0.5 mM EDTA, 1 mM DTT, 0.01% Tween-20). Proteolytic cleavage of the N-terminal GST tag was performed by incubation with PreScission Protease (GE Healthcare) overnight at 4 °C. The following day, the supernatant of the cleavage reaction was collected and incubated with fresh Glutathione Sepharose for 1 h at 4 °C to remove residual GST-tagged protease and uncleaved protein. Proteins were further purified against the C-terminal His tag by applying the supernatant onto washed Ni-NTA Profinity Resin (BioRad) and bound for 45 min at 4 °C. The Ni-NTA resin was subsequently washed three times in buffer containing 50 mM NaH_2_PO_4_ pH 8.0, 300 mM NaCl, 20 mM imidazole before eluting bound His-tagged buffer in the same buffer containing 250 mM imidazole. Protein eluates were concentrated using 0.5 ml centrifugal filter units (Merck Millipore) with a 10 kDa MWCO. Protein was quantified using Quick Start Bradford Reagent (BioRad). Protein purity was evaluated with Page Blue colloidal coomassie staining solution (Thermo Scientific). All *in vitro* binding experiments utilized freshly purified Tau protein immediately following purification.

### Co-Immunoprecipitation of Tau and synaptic vesicles

In this co-IP assay, recombinant Tau-His was used as bait to pull-down intact synaptic vesicles prepared from mouse brains. Briefly, 1 μg of freshly purified recombinant Tau-His protein was immobilized to Protein G Magnetic Dynabeads (Invitrogen) using anti-His antibodies (Invitrogen, clone 4A12E4) in vesicle binding buffer supplemented with 1% BSA for 2 h at 4 °C. The Tau-bound Dynabeads were then washed and incubated with mouse crude synaptic vesicles (LP2 fraction, equivalent to 20 μg of protein) in synaptic vesicle binding buffer supplemented with 1% BSA, 0.1% NP-40, 0.1% Tween-20, and protease inhibitors in a 500 μl reaction mixture for 2 h at 4 °C. Note that this low-concentration of detergents increases specificity of binding without lysing synaptic vesicles. The Dynabeads were thoroughly washed after incubation, and the bound proteins were retrieved by boiling in LDS containing 1% β-mercaptoethanol and assessed for the presence of synaptic vesicle marker proteins by immunoblotting. For the competition experiment with full-length Tau and Tat-NT^Tau^, intact synaptic vesicles (equivalent to 20 μg of protein) were immobilized on Dynabeads using 1 μg anti-Synaptobrevin 2 antibodies (Synaptic Systems, clone 69.1) for 4 h at 4 °C. After 4 hours, SV-bound Dynabeads were washed and then resuspended in vesicle binding buffer containing 1 μg (40 nM) of freshly purified full-length Tau-His with varying concentrations of freshly purified Tat-NT^Tau^-His. Reactions were left mixing at 4 °C for 2 h, after which beads were thoroughly washed and then boiled in LDS and assessed for the amount of full-length Tau bound to synaptic vesicles by immunoblotting.

### EM analysis of Tau-vesicle binding *in vitro*

To assess the binding of recombinant Tau-His to ultrapure rat synaptic vesicles, 1 μM Tau-His and 50 μg ml^−1^ vesicles were incubated together in synaptic vesicle binding buffer containing 1% BSA for 2 h at 4 °C in a 100 μl binding reaction. After binding, 2 μl of the vesicle suspension was dotted onto a glow-discharged, carbon-coated grid and let air dry. After drying, grids were blocked in 1% BSA for 5 min followed by incubation with 20 nM of 5 nm Ni-NTA-Nanogold (Nanoprobes) for 30 min in SV buffer containing 1% BSA. Grids were subsequently washed in SV buffer containing 8 mM imidazole before fixation in 2% glutaraldehyde for 5 min. Grids were then washed in ultrapure water before negative staining with 1% uranyl acetate. Samples were imaged on a Jeol JEM1400 transmission electron microscope.

### *In vitro* binding of phospho-Tau to synaptic vesicles

Recombinant human Tau (2N4R isoform, 441aa) was purified from bacterial culture (Tebu-Bio) and introduced into an *in vitro* phosphorylation reaction with human GSK3-β and CDK5 kinases purified from insect cells (Merck Millipore) in buffer containing 40 mM Tris-HCl (pH 7.2), 150 mM NaCl, 0.2% n-octyl-glucoside, 5 mM MgCl_2_, 1 mM EDTA, 2% glycerol, 5 mM ATP, 0.25 mg ml^−1^ Ovalbumin (GE Healthcare), 1 mM DTT and 0.1 mM Na_2_VO_4_. The reaction was allowed to proceed for 7 h at 35°. After 7 h, pure phosphorylated Tau was separated away from kinases and Tau degradation products by size exclusion chromatography on a Superdex-200 column. pTau-containing fractions were pooled, concentrated and stored at −80° for later use. Equal purity of Tau and pTau was confirmed by colloidal coomassie staining. To test the synaptic vesicle binding affinity of pTau, we thawed aliquots of Tau and pTau and pre-cleared aggregates at 100,000 *g* for 1 h. The supernatant was collected, protein concentration determined, and 500 ng of Tau or pTau was introduced into the sedimentation assay with synaptic vesicles prepared from mouse brains. Tau bound to vesicles was detected by immunoblotting the synaptic vesicle pellet with anti-total Tau (DAKO, 1:1000) antibodies.

### Immunofluorescence labelling of rat hippocampal neurons

Rat autaptic hippocampal neuronal cultures were transduced with AAV expressing Tau variants. At DIV 11, the cells were fixed in 4% paraformaldehyde+4% sucrose in PBS for 25 min at room temperature. After fixation, coverslips were washed for three times with PBS and incubated with blocking buffer (PBS containing 5% goat serum, 3% BSA and 0.3% Triton-X) for 1 h at room temperature. The coverslips were then incubated with primary antibodies overnight at 4 °C. Both primary and secondary antibodies were diluted in buffer PBS containing 1% goat serum, 2% BSA and 0.1% Triton-X. The next day, the coverslips were washed for three times with PBS and then incubated with secondary antibodies for 1 h at room temperature. After washing for five times with PBS, the immunolabled coverslips were mounted on glass slides with Vectashield mounting medium (Vector laboratories). The following antibodies were used: mouse anti-HA (Covance; 1:500), rabbit anti-Synapsin (Merck Millipore; 1:500) and chicken anti-MAP2 (abcam; 1:5000), Alexa-555 goat-anti-mouse, Alex-647 goat-anti-chicken and Pacific Blue goat-anti-rabbit (Invitrogen; 1:5000). All digital images were captured using a Nikon confocal microscope with a × 60 oil lens, and were further processed using Image J software. For quantification of the images, rectangles with area of ∼2,500 μm^2^ containing distal axons were randomly selected, and the numbers of Tau/Synapsin dual-positive puncta and Synapsin-positive puncta within the selected areas were counted manually.

### Histology of fly brains

Adult fly heads were fixed in PBS containing 4% formalin for 4 h at room temperature. After fixation, samples were dehydrated in ethanol series and embedded in paraffin overnight. A microtome (Leica RM2135) with a disposable knife was used to make frontal sections of the adult heads. A series of sections (5 μm) spanning the brain were collected on a glass slide and subject to H&E histology staining according to standard protocols. Stained sections were visualized using a microscope (LeicaDMRA2), and images were acquired with a digital camera (Photometrics, CoolSnap 5.0) steered by the Northern Eclipse 6.0 software (EMPIX Imaging Inc.).

### Phalloidin staining of hippocampal neurons

Primary hippocampal neurons were isolated (described above) and plated at density of 140 cells mm^−2^ on poly-L-lysine coated coverslips. Neurons were transduced (at DIV 5) with bicistronic AAV virus co-expressing Tau and GFP (to indicate transduced neurons), or transduced with control AAV virus expressing GFP alone. At DIV 11, transduced neurons expressing Tau_P301L were pretreated with 5 μM Tat-NT^Tau^ for 4 h. Neurons were fixed in 4% paraformaldehyde, 4% sucrose in PBS and blocked with blocking solution (5% goat serum, 3% BSA, 0.3% Triton-X-100 in PBS). The coverslips were incubated with rabbit anti-Synaptophysin I antibody (Synaptic Systems, 1:1,000) in incubation buffer (1% goat serum, 2% BSA, 0.1% Triton-X-100 in PBS) overnight at 4 °C. The next day, the coverslips were washed and incubated with anti-rabbit Pacific Blue secondary antibodies (Life Technologies, 1: 5,000) and 1 μg ml^−1^ TRITC-Phalloidin (Sigma) for 1 h at room temperature, followed by washing and mounting. Synaptophysin-labelled punctae along the axon were used to indicate presynaptic compartments. The amount of presynaptic F-actin was quantified by measuring the Phalloidin fluorescence intensity (integrated density) within hand-drawn ROIs around Synaptophysin-labelled punctae using ImageJ.

### Statistics

The results in graphs depicted in dot plots are shown as average±s.e.m. unless otherwise noted. Statistical testing was performed using GraphPad and SigmaPlot 12.3 (Systat Software Inc), Student *t*-test, One-Way ANOVA and Two-Way ANOVA. The significance levels are indicated by asterisks; *P<0.05; **P<0.01; ***P<0.001.

### Data availability

The data that support the current findings are available from the corresponding author on request.

## Additional information

**How to cite this article:** Zhou, L. *et al*. Tau association with synaptic vesicles causes presynaptic dysfunction. *Nat. Commun.* **8,** 15295 doi: 10.1038/ncomms15295 (2017).

**Publisher's note:** Springer Nature remains neutral with regard to jurisdictional claims in published maps and institutional affiliations.

## Supplementary Material

Supplementary Information Supplementary Figures, Supplementary Table, Supplementary Methods and Supplementary References
