# Supplementary material for: Tau association with synaptic vesicles causes presynaptic dysfunction
Source: Nat Commun. 2017 May 11;8:15295. doi: 10.1038/ncomms15295 (PMC5437271; doi:10.1038/ncomms15295)
Supplement: Supplementary Information — Supplementary Figures, Supplementary Table, Supplementary Methods and Supplementary References [file ncomms15295-s1.pdf]

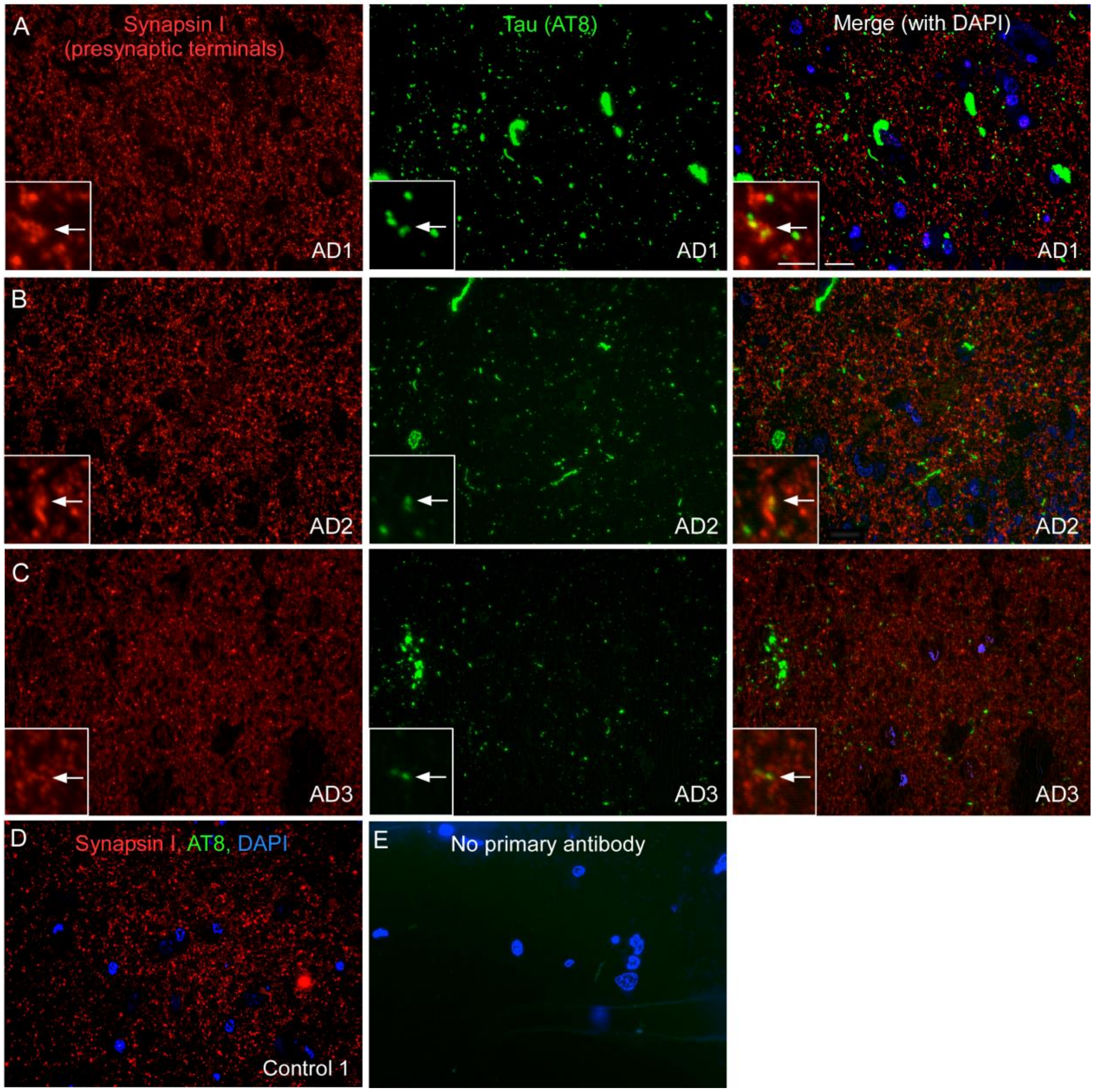

Supplementary Figure 1. Pathologically phosphorylated Tau is localized to the presynaptic terminals of Alzheimer's disease (AD) patient brains. Ultrathin (70 nm) sections of healthy control or AD patient brains were analyzed by array tomography. Presynaptic terminals were labeled with anti-Synapsin I antibody and phospho-Tau was labeled with AT8 antibody. Immunostainings show AT8-positive Tau at presynaptic terminal (arrows) of AD patient brain (A-C) but not in control patient brain (D), while negative control staining showed no background staining from secondary antibodies (E). Presynaptic phospho-Tau was observed in all three AD cases examined but not in any of the four healthy control cases examined. See Table S1 for patient case information. Scale bar: 10 µm, 2µm (inset).

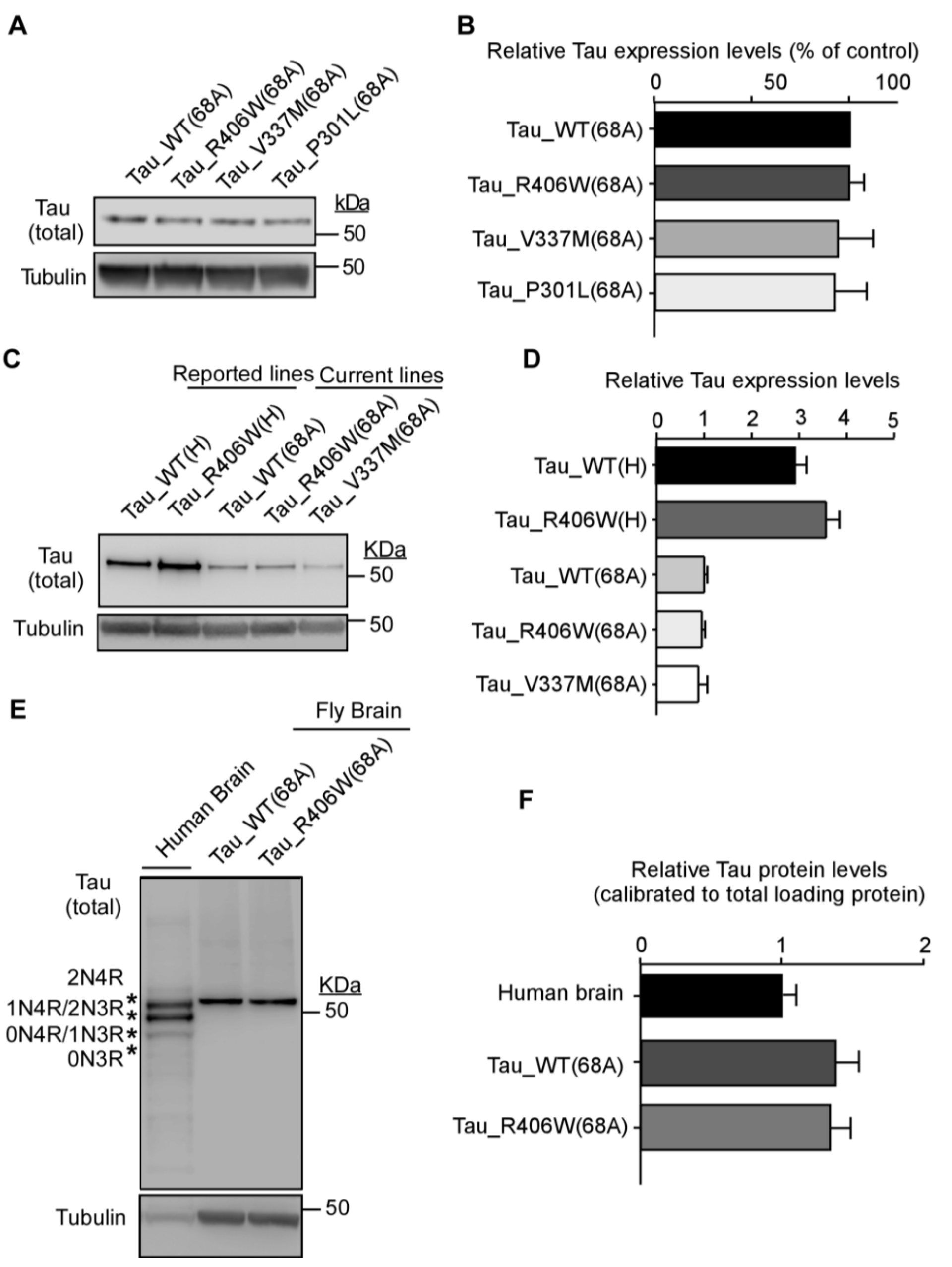

Supplementary Figure 2. Relative Tau expression levels of the UAS-Tau fly lines used in the current work as compared to that of previously reported fly lines and human brains. (A-B) Relative Tau expression levels in the brains of UAS-Tau transgenic fly lines. (A) Representative immunoblot detects total Tau expression under the control of the D42-Gal4 motor neuron-specific driver (with DAKO antibody); Tubulin was used as a loading control. (B) Quantification of Tau expression from immunoblots shows similar expression levels of Tau variants in the brains of tested transgenic lines (n= 3 independent experiments). **(C-D)** The UAS fly lines Tau_WT (H) and Tau_R406W(H) were used in the previous work (see Methods in the main text), whereas Tau_WT(68A), Tau_R406W(68A) and Tau_V337M(68A) are from the current work. Representative immunoblot detects total Tau (with DAKO antibody) and Tubulin (loading control) from fly brains (C) and quantification of the immunoblots (D) shows that the current fly lines from this work express 3-to-4 fold lower levels of Tau as compared to the previously reported fly lines (n =4 independent experiments). **(E-F)** Human entorhinal cortex brain sample was obtained from MRC London brain bank. Equal amount of total protein lysates (15 µg) from human brain and fly brains were loaded

on electrophoresis gels and analyzed by immunoblotting for total Tau (with DAKO antibody) and Tubulin (E) and quantification of the immunoblots (F) shows that the expression of human Tau in fly brains is similar to the Tau expression level in the human brain (0N4R/1N3R band was used for quantification). Note that 1) Tau (0N4R) from fly brains shows slightly slower electrophoretic mobility compared to that from human brain, this is probably caused by variations in specific post-translational modifications of Tau in fly and human brains; and 2) Despite the equal loading of total protein, Tubulin staining shows higher intensity in fly brain samples as compared to human brain sample, this is likely caused by higher antibody affinity to fly Tubulin than to human Tubulin. However, the quantification (F) is not calibrated to Tubulin level but is based on the equal amount of total protein use for loading (n =3 independent experiments).

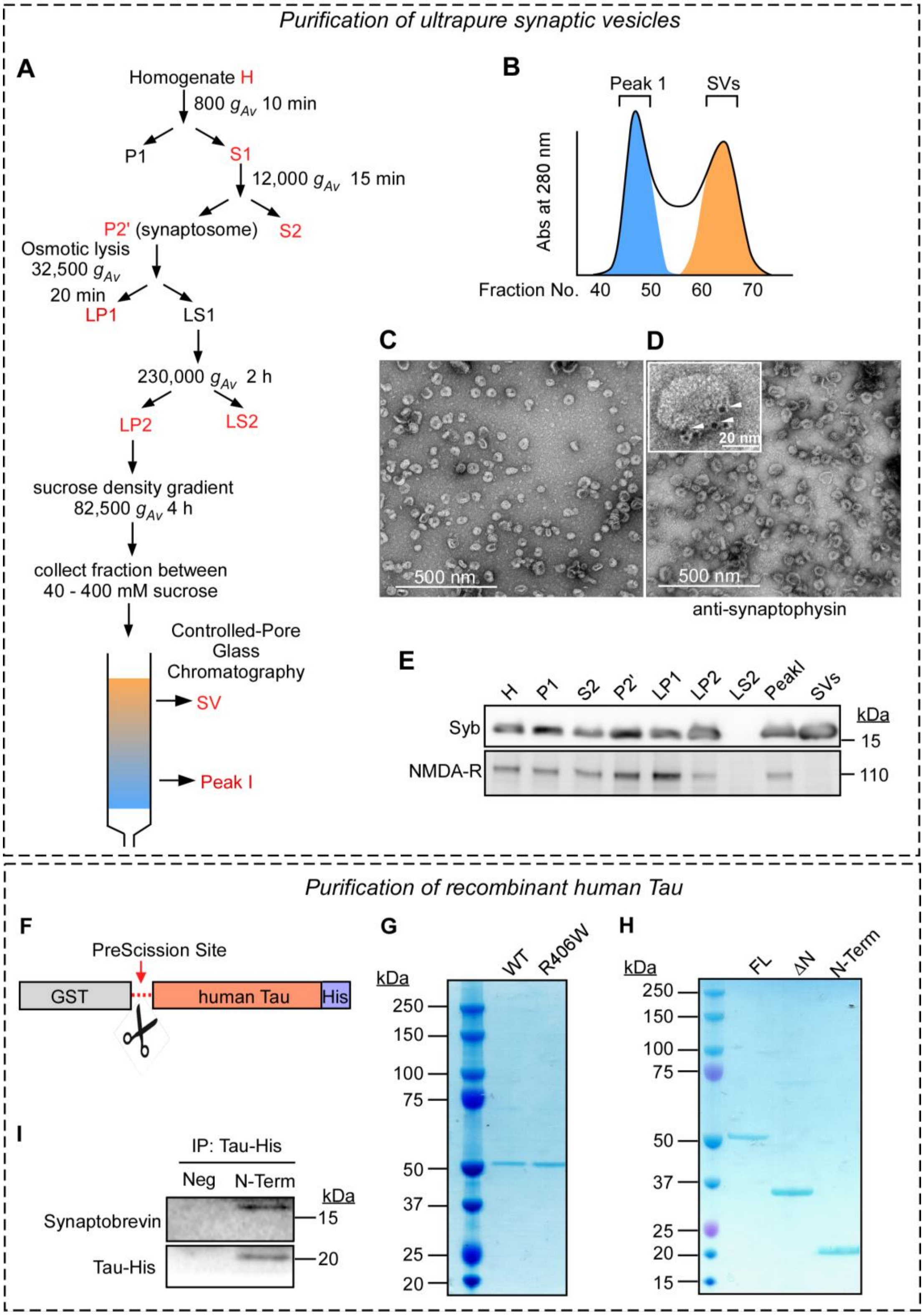

Supplementary Figure 3. (A-E) Preparation of ultrapure synaptic vesicles from rat brains using controlled-pore glass chromatography. Schematic graph (A) shows the procedure of preparing ultrapure synaptic vesicles (SVs) from rat brain by differential centrifugation, followed by controlled-pore glass chromatography (B) in which

membrane debris elute in fraction “Peak 1” followed by ultrapure vesicles in the second peak, or “SV” fraction. Fractions containing SVs (~60-70) are then combined and concentrated by centrifugation. See Materials and Methods for complete description. **(C-E)** Quality control of the ultrapure synaptic vesicles. Negative-stain electron microscopy (EM) shows a homogenous population of small vesicles in the size range of 40 to 50 nm (C); Immuno-EM shows that more than 95% of vesicles are positive for the synaptic vesicle marker synaptophysin (D); Immunoblotting of the fractions collected from the fractionation procedure shows the enrichment of a synaptic vesicle marker protein Synaptobrevin and depletion of a post-synaptic marker protein NMDA-receptor in the final synaptic vesicle fraction (E). **(F-H) Purification of recombinant human Tau from bacterial cultures**. **(F)** Schematic depicting purification of GST/His double-tagged Tau from bacterial cultures, and proteolytic removal of the N-terminal GST tag by PreScission protease to result in the final product Tau-His (See Materials & Methods). **(G-H)** Colloidal coomassie staining of purified full-length WT and R406W Tau-His$^{1\text{-}383}$ (M), N-terminally truncated Tau$^{113\text{-}383}$-His (ΔN) and N-terminal fragment (Tau$^{1\text{-}112}$-His) recombinant proteins (N) used for *in vitro* synaptic vesicle binding assays. **(I) The N-terminal domain of Tau is sufficient to bind synaptic vesicles.** Representative immunoblot detects Tau$^{1\text{-}112}$-His (N-Term) and the synaptic vesicle marker Synaptobrevin from co-IP using anti-His antibodies.

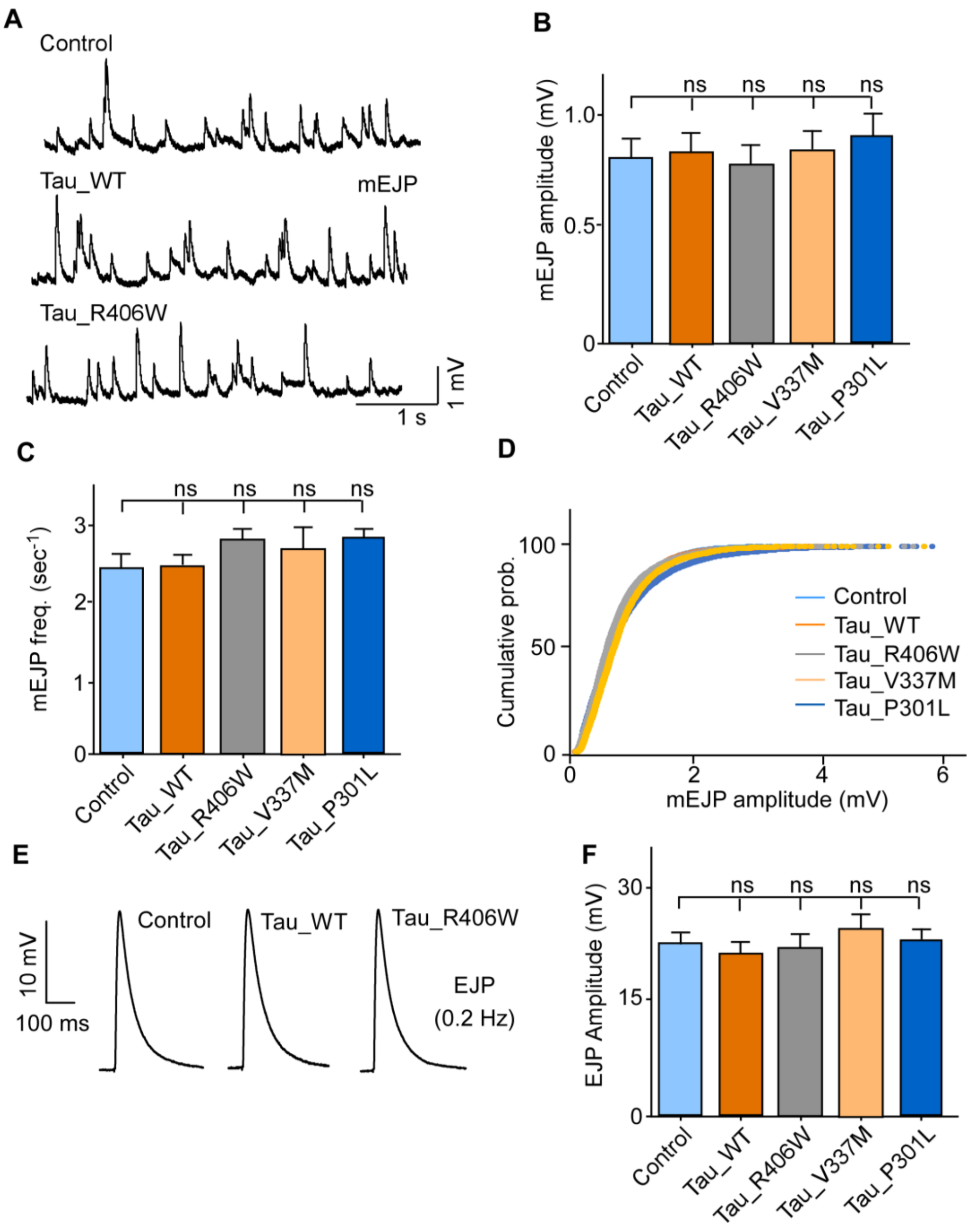

Supplementary Figure 4. Tau does not affect basal neurotransmitter release. Electrophysiological recordings were performed at the NMJs of *Drosophila* larvae expressing UAS-Tau (WT, R406W, V337M or P301L) under the D42-Gal4 motor neuron driver. **(A-D)** Representative spontaneous miniature excitatory junctional potential (mEJP) traces (A) and the plot of mEJP amplitude (B), mEJP frequency (C) and cumulative probabilities (D). One-way ANOVA, ns, not significant, mean ± SEM, n=10-12 NMJs (≥5 animals). **(E-F)** Representative evoked junction potential (EJP) traces in response to stimulation at 0.2 Hz (E), and Plot of EJP amplitudes averaged during 1 minute of stimulation at 0.2 Hz (F). One-way ANOVA, ns, not significant, mean ± SEM, n=7-9 NMJs (≥5 animals).

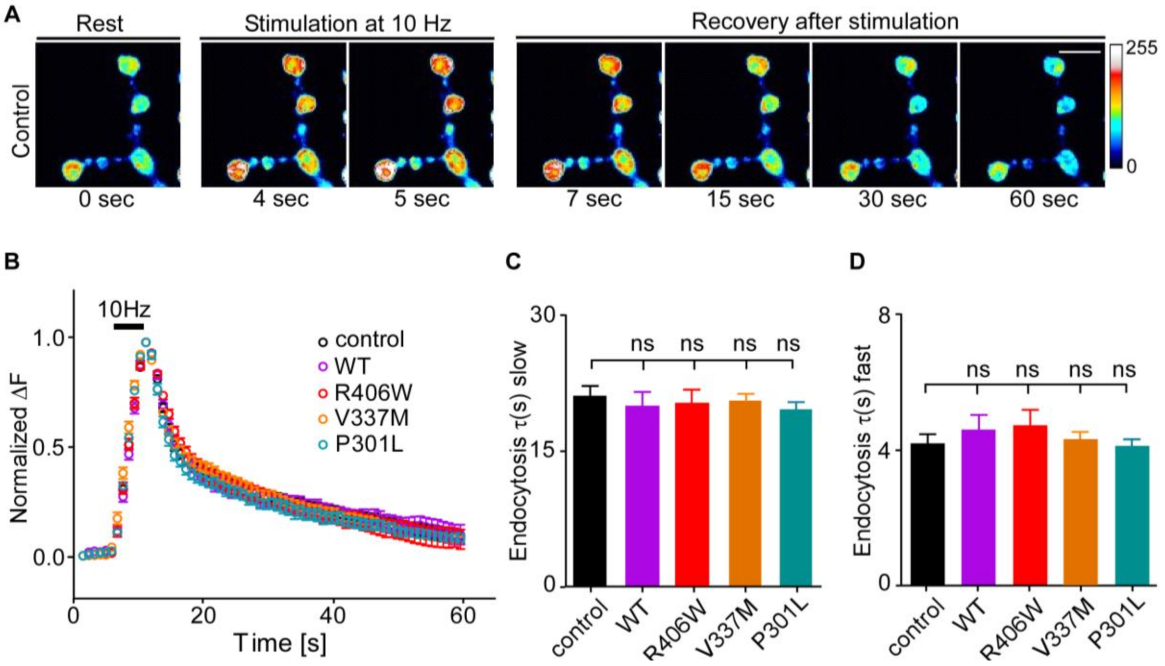

Supplementary Figure 5. Tau does not affect endocytosis in the Synapto-pHluorin (SpH) assay. *Drosophila* larvae used in this assay co-express UAS-SpH and UAS-Tau (WT, R406W, V337M or P301L) under D42-Gal4 motor neuron driver. (A) Representative images show the typical SpH response of NMJ synapses upon short stimulation at 10 Hz for 5 seconds. (B-D) Plot of fluorescence change (ΔF) (B) normalized as ratio to peak ΔF. To assess endocytosis rates, fluorescence decays after stimulation were fitted in two-phase decay curves and the time constants for both slow (C) and fast (D) decays were plotted, showing no significant difference in endocytosis between control and Tau-expressing animals. One-way ANOVA, ns, not significant, mean ± SEM, n=7-12 NMJs (≥5 animals). Scale bar, 10 µm.

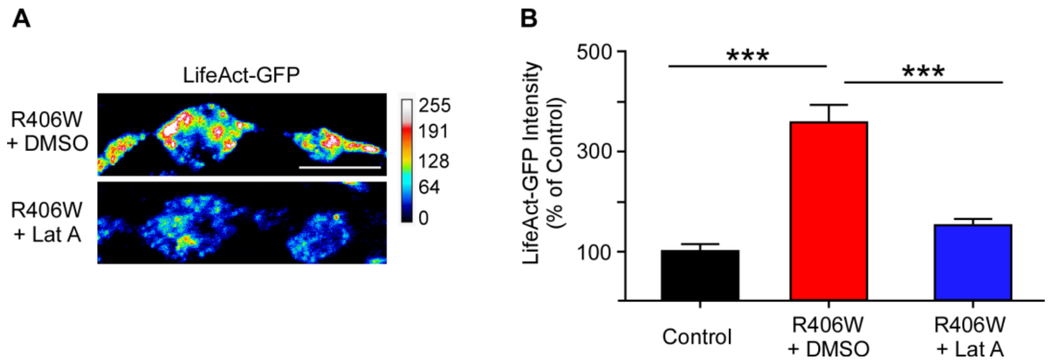

Supplementary Figure 6. Treatment with the F-actin depolymerizing drug Latrunculin A reduces presynaptic F-actin levels at *Drosophila* NMJs. *Drosophila* larvae express UAS-LifeAct-GFP and/or UAS-Tau_R406W under the D42-Gal4 motor neuron driver. Larvae were bathed in 10 µM Lat A for 7-0 minutes then fixed and immunostained for GFP. Polymerized F-actin levels were measured by measuring LifeAct-GFP fluorescence intensity. **(A)** Representative images of LifeAct-GFP signal in larvae expressing Tau_R406W following treatment with either DMSO or LatA. **(B)** Quantification of LifeAct-GFP intensity of larvae expressing D42>LifeAct-GFP (control) or coexpressing LifeAct-GFP and Tau_R406W with DMSO or Lat A treatment. ***P<0.001. One-way ANOVA, n=14-20 NMJs (≥5 animals). Scale bar, 5 µm.

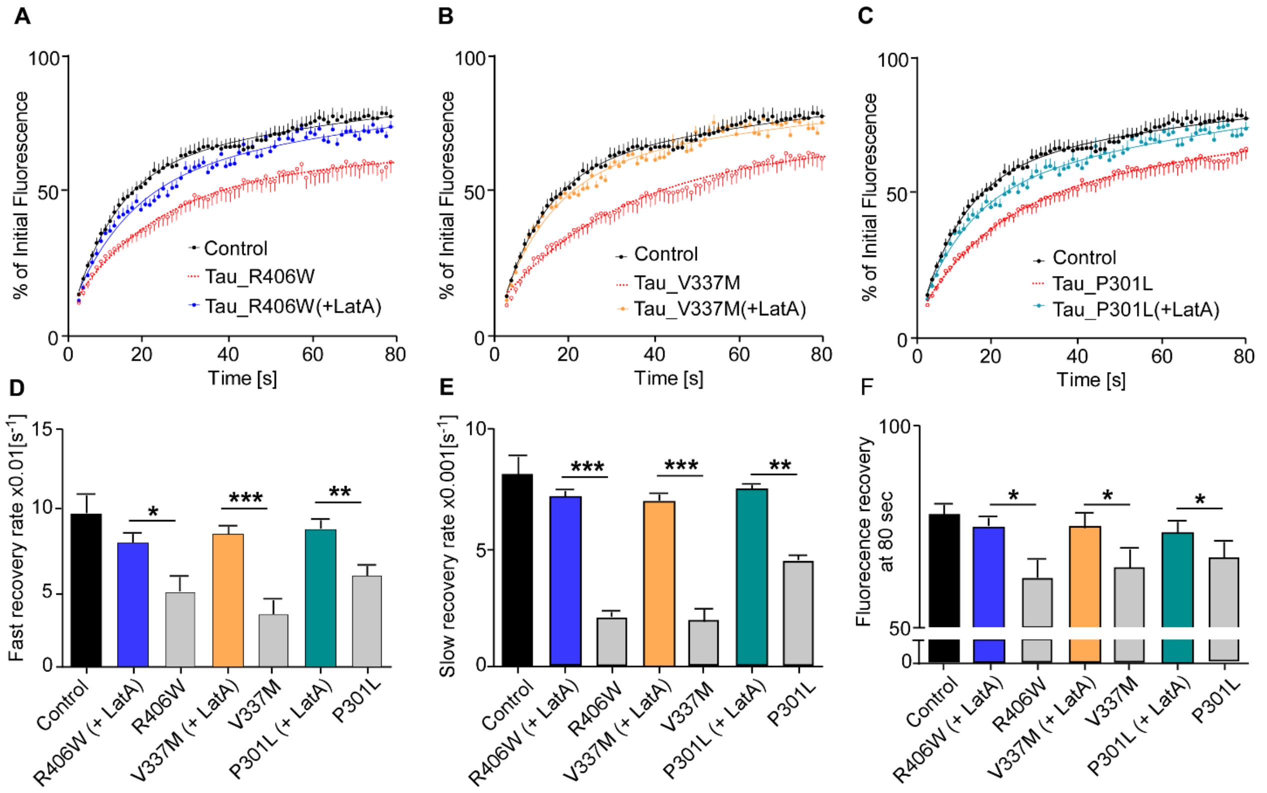

Supplementary Figure 7. Treatment with the F-actin depolymerizing drug Latrunculin A rescues pathogenic mutant Tau-induced vesicle mobility deficits at *Drosophila* NMJs. *Drosophila* larvae coexpress UAS-Tau (R406W, V337M or P301L) and UAS-Synaptotagmin under the D42-Gal4 motor neuron driver. Fluorescence-recovery-after-photobleaching (FRAP) measurements of vesicle mobility within synaptic boutons were performed after treatment of Latrunculin A. **(A-C)** Plots of fluorescence recovery (% of initial fluorescence) over time and fit with double-exponential curve. **(D-E)** Plots of fast and slow recovery rates calculated from fluorescence recovery curve. **(F)** Plot of fluorescence recovery at time=80 sec. * P<0.5, **P<0.01, ***P<0.001. Student's *t-test*, n=21-25 boutons (≥5 animals).

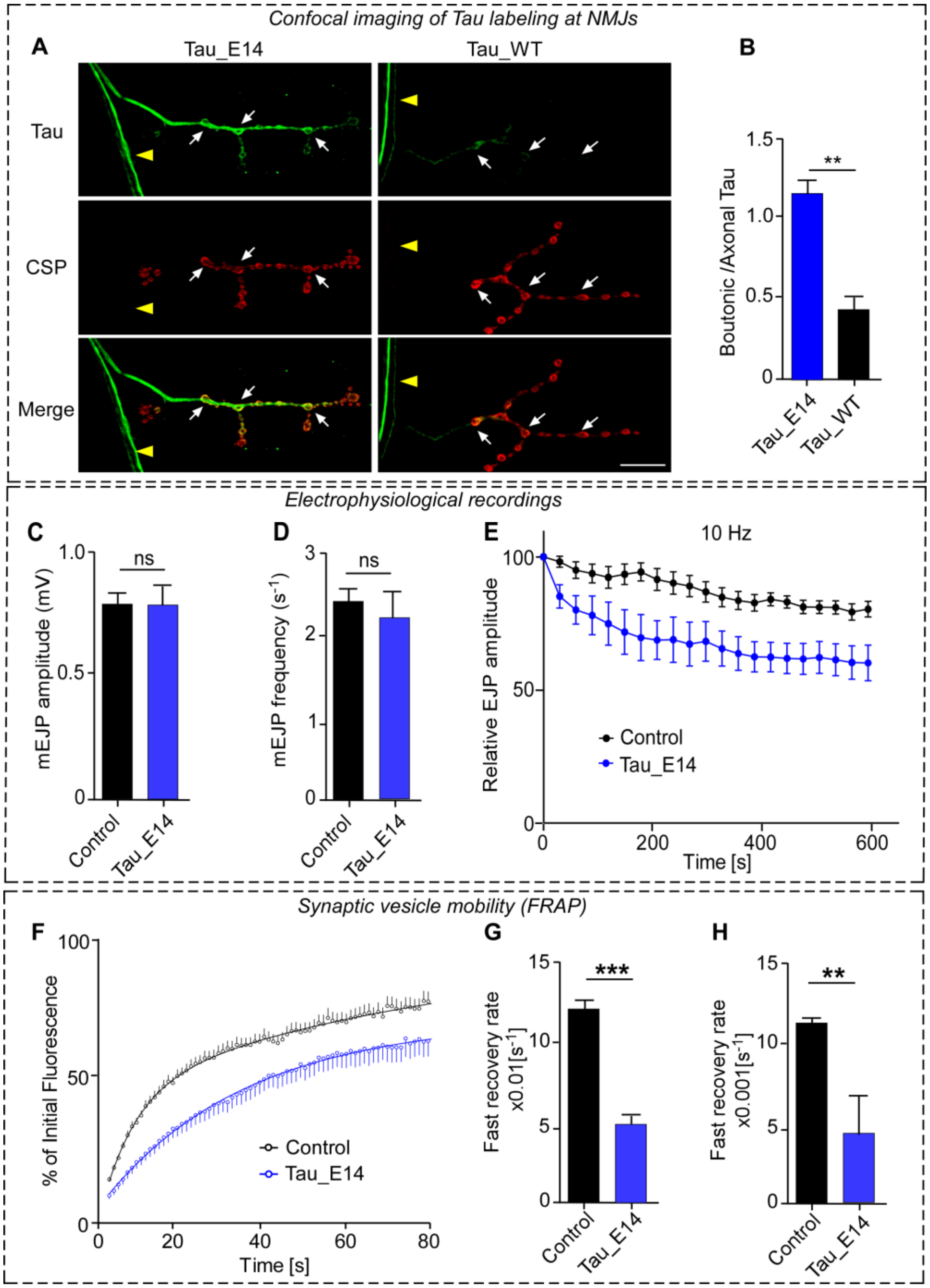

Supplementary Figure 8. Phosphomimetic mutant Tau is localized to presynaptic terminal and affects synaptic vesicle dynamics. *Drosophila* larvae used in these assays express UAS-Tau_E14 (A-E) or co-express UAS-Tau_E14 and UAS-synaptotagmin (F-H) under the D42-Gal4 motor neuron driver. **(A-B)** Confocal imaging of Tau and CSP immunolabeling at NMJs (A) and quantification of Tau fluorescence intensity within synaptic boutons as ratio to axonal Tau fluorescence intensity (B). Synaptic boutons (arrows) and axons (arrowheads) are indicated. **P<0.01, Student's *t test*, n=8-11 NMJs (≥4 animals). Scale bar, 20 nm. **(C-E)** Electrophysiological recordings at NMJs. Plot of spontaneous miniature excitatory junctional potential (mEJP) amplitude (C) and mEJP frequency (D). Student's *t-test*, ns, not significant, n=10 NMJs (≥5 animals). Plot of relative evoked junctional potential (EJP) during 10 min stimulation at 10 Hz (E). n=7 NMJs (7 animals). **(F-H)** Fluorescence-recovery-after-photobleaching measurement of vesicle mobility within synaptic boutons at NMJs. Plot of fluorescence recovery (% of initial fluorescence) over time and fit with double-exponential curve (F). The fast (G) and slow (H) recovery rates are calculated from fluorescence recovery curves. Student's t-test, n=22-25 boutons (≥5 animals). * P<0.5, **P<0.01, ***P<0.001, ns, not significant.

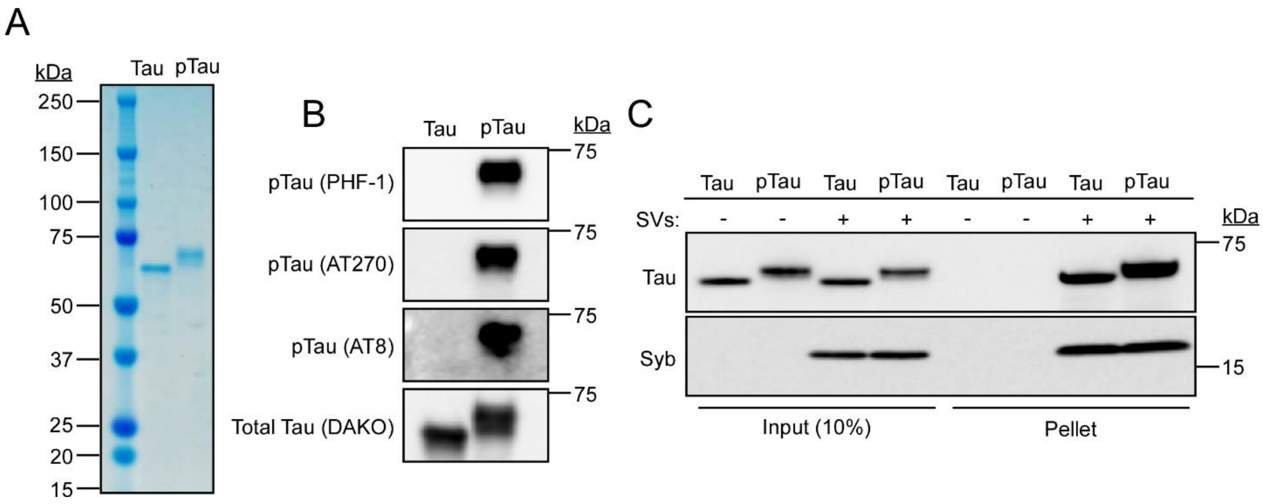

Supplementary Figure 9. Phosphorylation of Tau does not affect its binding affinity to synaptic vesicles. (A) Colloidal coomassie staining of purified Tau (2N4R isoform). Purified Tau was incubated together with GSK3-β and CDK5 kinases followed by size exclusion chromatography to yield pure phosphorylated Tau (pTau). (B) Western blotting confirms immuno-reactivity for phospho-Tau epitopes on pTau. (C) Sedimentation assay shows the equal ability of Tau and pTau (detected with Total Tau (DAKO) antibody) to bind and co-sediment together with synaptic vesicles (identified by SV marker Synaptobrevin-2, Syb). Western blot is representative of three experiments.

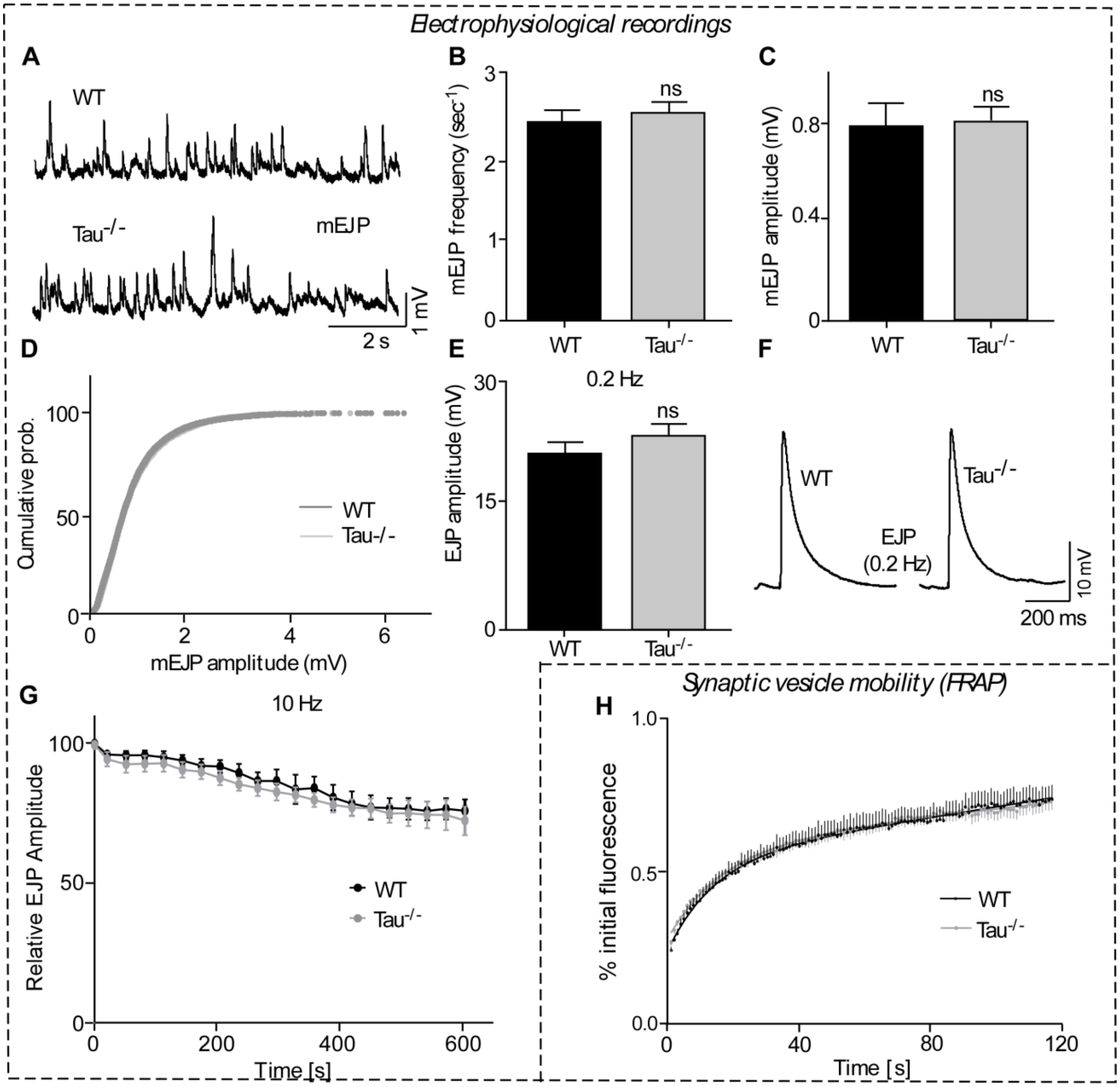

Supplementary Figure 10. (A-G) Tau knockout flies show normal neurotransmitter release at basal conditions and sustain synaptic transmission during high-frequency stimulations. Electrophysiological recordings were performed at the NMJs of *Drosophila* larvae. **(A-D)** Representative spontaneous miniature excitatory junctional potentials (mEJPs) (A), plot of mEJP amplitude (B), mEJP frequency (C) and cumulative probabilities (D). Student's t-test, ns, not significant, mean ± SEM, n=10-12 NMJs (≥5 animals). **(E-F)** Representative evoked junctional potentials (EJPs) in response to stimulation at 0.2 Hz (F), plot of mEJP amplitude (E). Student's t-test, ns, not significant, mean ± SEM, n= 6 NMJs (3 animals). **(G)** Plot of relative EJP amplitudes during 10 min stimulation at 10 Hz. n=7 NMJs (7 animals). **(H) Tau knockout flies show normal synaptic vesicle mobility in fluorescence-recovery-after-photobleaching (FRAP) assay.** Synaptic vesicles at *Drosophila* larvae NMJs were labeled with FM1-43 dye by KCl stimulation for FRAP measurement. Fluorescence recovery (% of initial fluorescence) over time were plotted and fitted with double-exponential curve. n=25 boutons (≥5 animals).

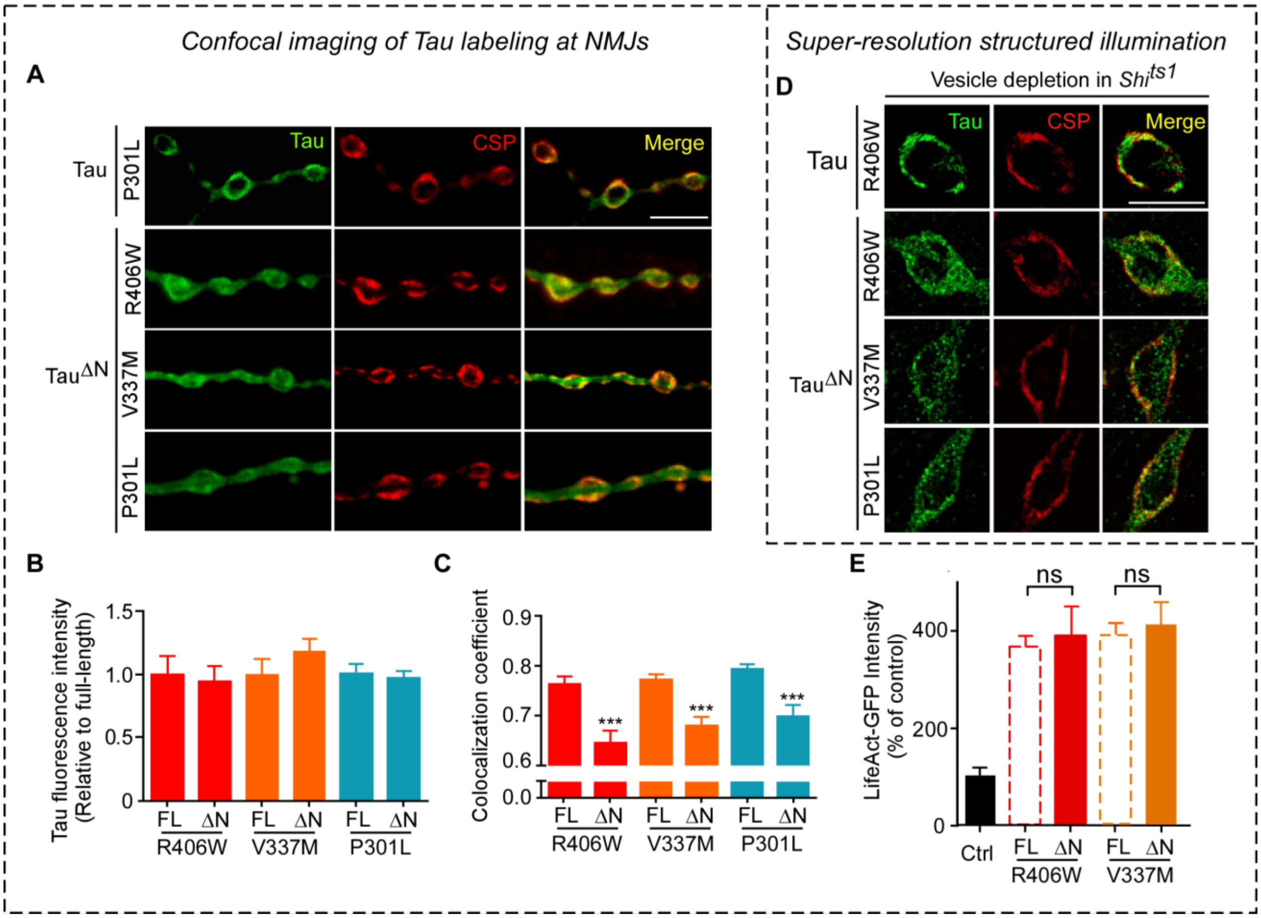

Supplementary Figure 11. (A-D) N-terminally truncated pathogenic mutant Tau localizes to presynaptic terminals of fly NMJs, but exhibits reduced association with synaptic vesicles *in vivo*. (A-C) *Drosophila* larvae used in these assays express UAS-Tau or UAS-Tau$^{\Delta N}$ (R406W, V337M or P301L) under control of the D42-Gal4 motor neuron driver. (A) Immunolabeling of Tau and CSP at NMJ synapses analyzed by confocal microscopy imaging. Scale bar, 10 µm. Quantification shows that full-length Tau and N-terminally truncated Tau are present at similar levels within synaptic boutons (B). However, N-terminally truncated Tau, as compared to full-length Tau, displayed reduced co-localization with the synaptic vesicle marker CSP as quantified using the Pearson's colocalization coefficient (C). Student *t-test*, ***P<0.005, n=20-30 NMJs (≥7 animals). (D) Super-resolution structured illumination microscopy analysis of Tau and CSP immunolabeling within SBs after depletion of synaptic vesicles in the *Shi*^{ts1} mutant background. Unlike full-length Tau which largely follows the re-localization of CSP to presynaptic membrane, the N-terminally truncated Tau remains within SBs to some extent, suggesting that N-terminally truncated Tau exhibits reduced association with synaptic vesicles *in vivo*. Scale bar, 5 µm. **(E) N-terminally truncated Tau increases presynaptic F-actin levels.** *Drosophila* larvae used in this assay co-express UAS-LifeAct-GFP and UAS-Tau or UAS-Tau$^{\Delta N}$ (R406W or V337M) under control of the D42-Gal4 motor neuron driver. Relative intensities of boutonic LifeAct-GFP probing for F-actin were plotted, showing that N-terminally truncated pathogenic mutant Tau (R406W or V337M) increases presynaptic F-actin levels similar to their full length counterparts. Student's t-test, n ≥ 12 NMJs (≥ 6 animals). Graphs in B and E depict mean, error bars are SEM. Graph C depicts mean, with whiskers depicting minimum and maximum.

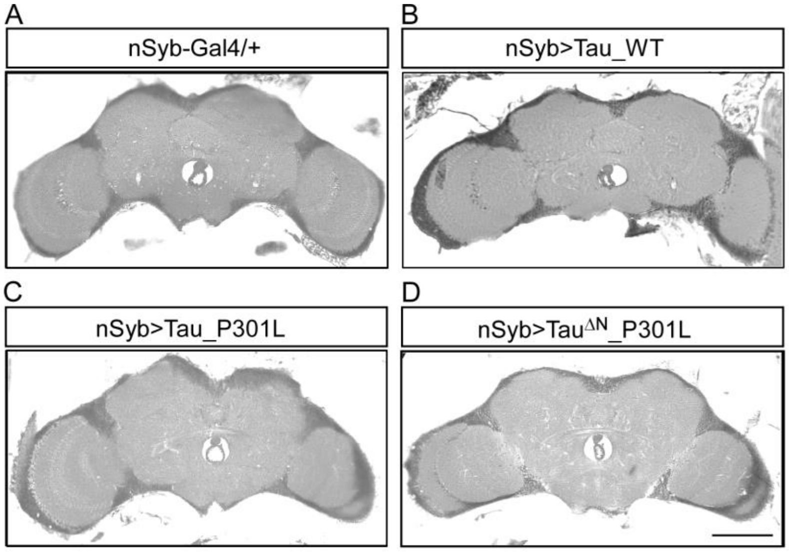

Supplementary Figure 12. Hematoxylin and Eosin (H&E) staining of frontal brain sections. Flies express UAS-Tau under a pan-neuronal driver nSyb-Gal4. No obvious vacuolar degeneration in the brains of nSyb-Gal4/+ (A), nSyb>Tau_WT (B), nSyb>Tau_P301L and nSyb> Tau$^{\Delta N}$_P301L (D) at 26-days of age. Scale bar, 100 µm.

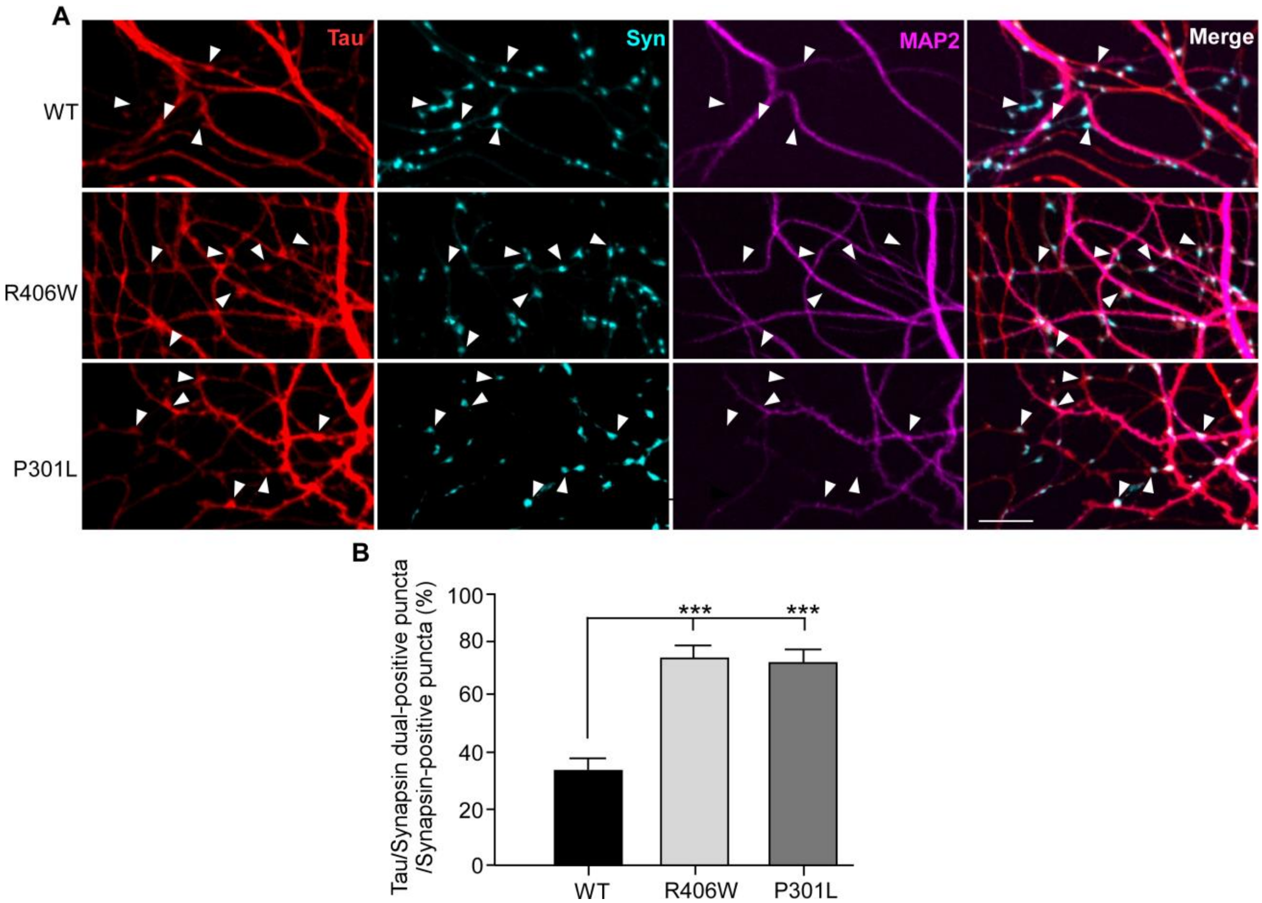

Supplementary Figure 13. **Localization of Tau to presynaptic terminals in rat hippocampal neurons.** Autaptic rat hippocampal neuronal cultures were transduced with AAV viral vectors expressing human Tau variants, and were fixed at DIV11-13 for immunolabeling. **(A)** Confocal images of rat neurons stained with antibodies against HA (detecting HA-tagged hTau), Synapsin (presynaptic marker) and MAP2 (dendritic maker). All Tau variants (WT, R406W and P301L) are presented in neurites, but mutant Tau (R406W, P301L) appear to be more punctate and colocalize with Synapsin-positive puncta. Arrow heads point to Synapsin-positive puncta. Scale bar, 10 µm. **(B)** Quantification of Tau- and Synapsin- dual-positive puncta in ratio to total Synapsin-positive puncta. A higher percentage of Synapin-positive puncta contain hTau in neurons expressing mutant Tau (R406W, P301L) as compared to neurons expressing WT Tau. n=23 (WT), 21 (R406W) and 19 (P301L) neurons from three independent cultures. One-way ANOVA, ***p<0.0001.

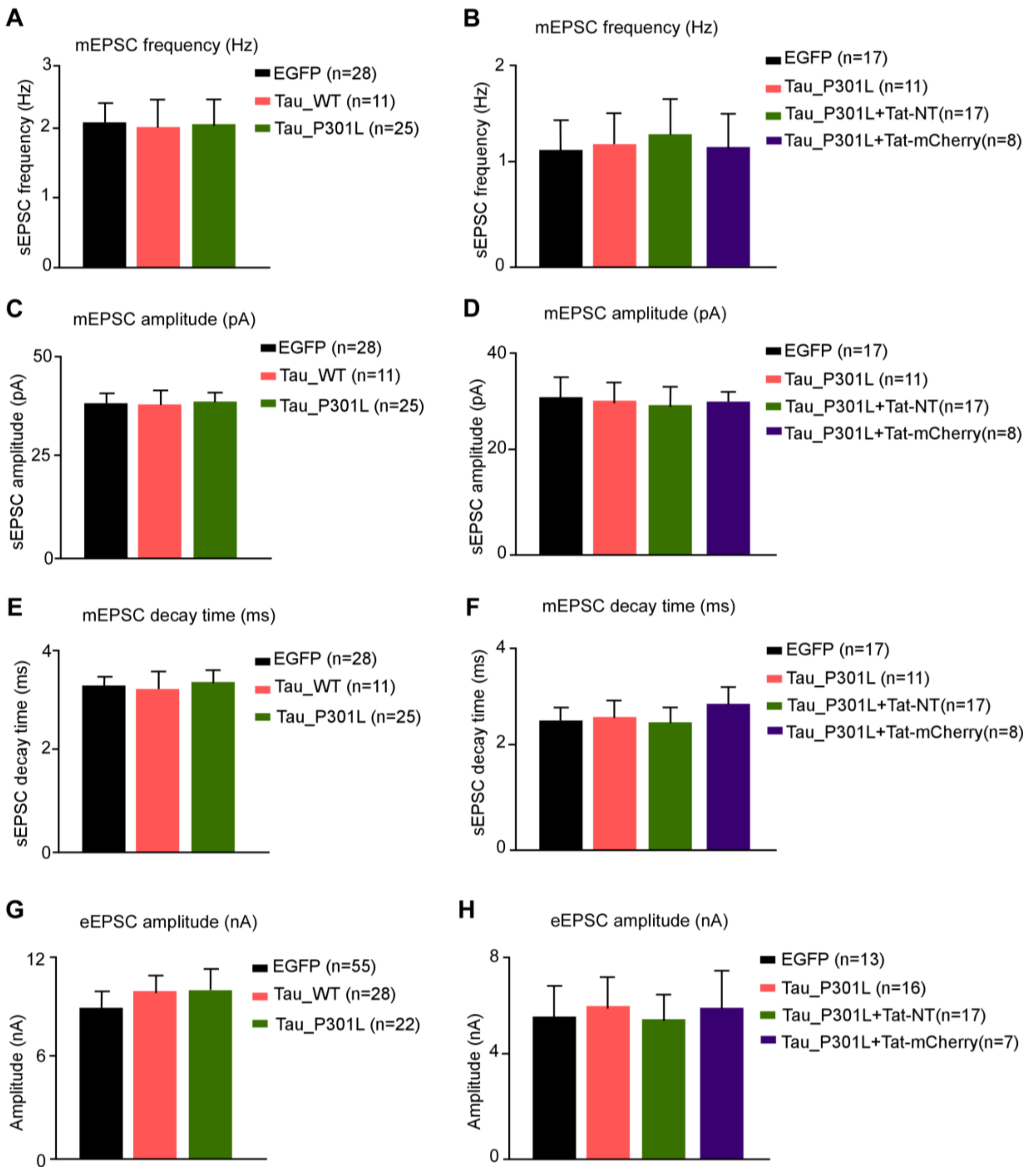

Supplementary Figure 14. Electrophysiological recordings of basal synaptic activity from rat autaptic culture of neurons. Autaptic neuronal cultures were transduced with AAV-EGFP, AAV-Tau_WT or AAV-Tau_P301L and acutely treated with 5 µM Tat-NT or Tat-mCherry (control) peptide. **(A-F)** The plots for spontaneous mEPSC frequency (A, B), mEPSC amplitude (C, D) and mEPSC decay time (E, F). **(G-H)** First evoked EPSC amplitude. The numbers of cells used for recording are indicated in the graphs; measurements were from at least three independent experiments. Mean ± SEM. One-way ANOVA, no significant difference between tested genotypes and control.

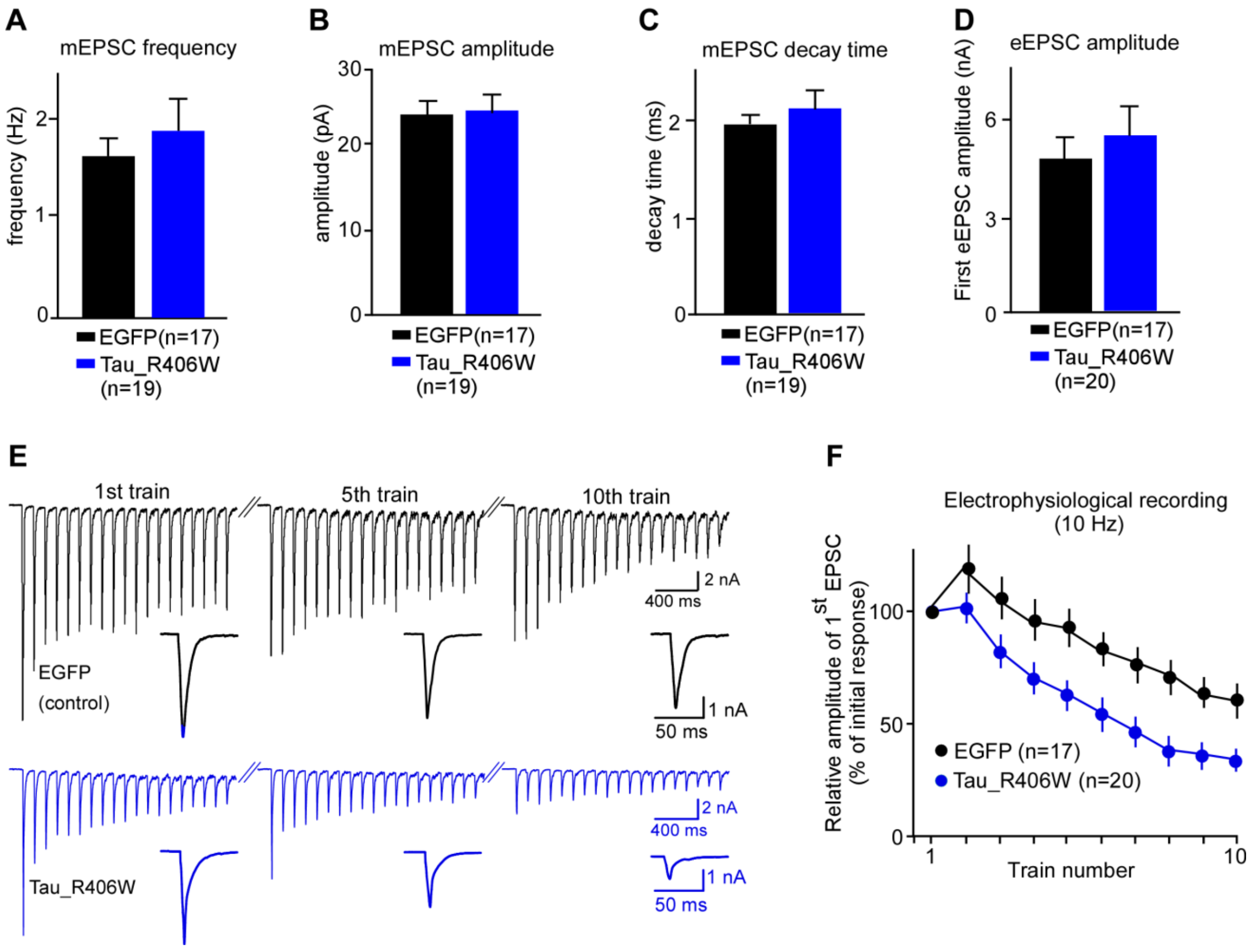

Supplementary Figure 15. Pathogenic mutant Tau (R406W) impairs neurotransmitter release during sustained high frequency stimulation trains in cultured rat hippocampal neurons. Autaptic rat hippocampal neuronal cultures were transduced with AAV viral vectors expressing GFP or Tau_R406W. **(A-C)** Electrophysiological recordings of spontaneous miniature excitatory postsynaptic currents (mEPSCs). The mEPSC frequency (A), amplitude (B) and decay time (C) are plotted. **(D-F)** Electrophysiological recordings of evoked excitatory postsynaptic currents (EPSCs) using patch clamp in response to 10 consecutive high frequency stimulation trains (10 Hz for 10 s with 30 s interval). The first EPSCs amplitude (D) of the initial stimulation train is plotted. The representative traces are shown in (E) and the relative first EPSCs were plotted to train numbers (F). The numbers of cells used for recording are indicated in the graphs; measurements were from at least three independent experiments.

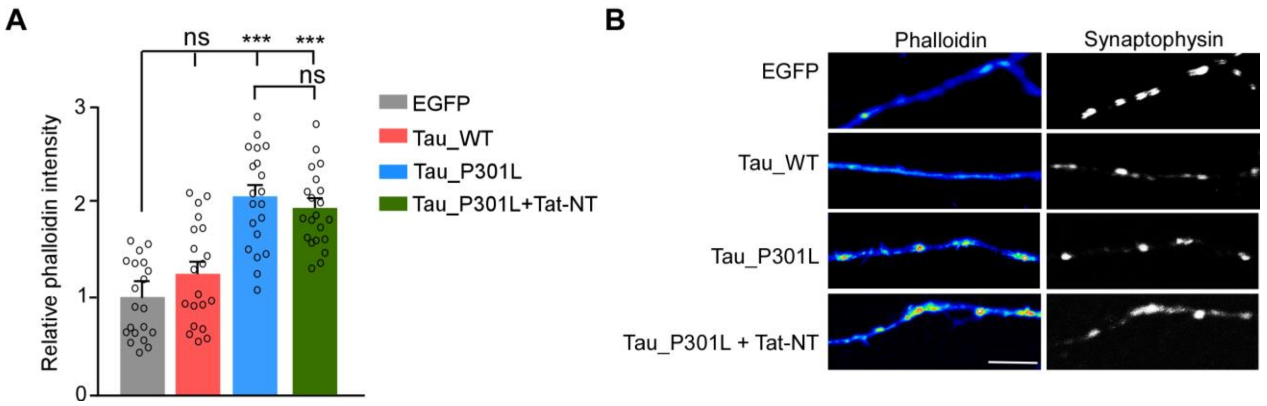

Supplementary Figure 16. Presynaptic F-actin staining of primary cultured hippocampal neurons. Primary cultured hippocampal neurons were transduced with AAV-EGFP, AAV-Tau_WT or AAV-Tau_P301L. Neurons transduced with AAV-Tau_P301L were acutely treated with 5 µM Tat-NT^{Tau} peptide for 4 hours. At DIV 11, neurons were fixed and stained with anti-Synaptophysin I antibody and TRITC-Phalloidin. The amount of pre-synaptic F-actin was determined by measuring the Phalloidin fluorescence intensity within Synaptophysin-labeled punctae. (A) Graph of relative Phalloidin fluorescence intensity within Synatophysin-labeled punctae. The numbers of cells used for quantification are n=20 (EGFP, Tau_WT, Tau_P301L) and n=21 (Tau_P301L+Tat-NT^{Tau}). (B) Representative staining for Synaptophysin and TRITC-Phalloidin. Scale bar, 10 µm.

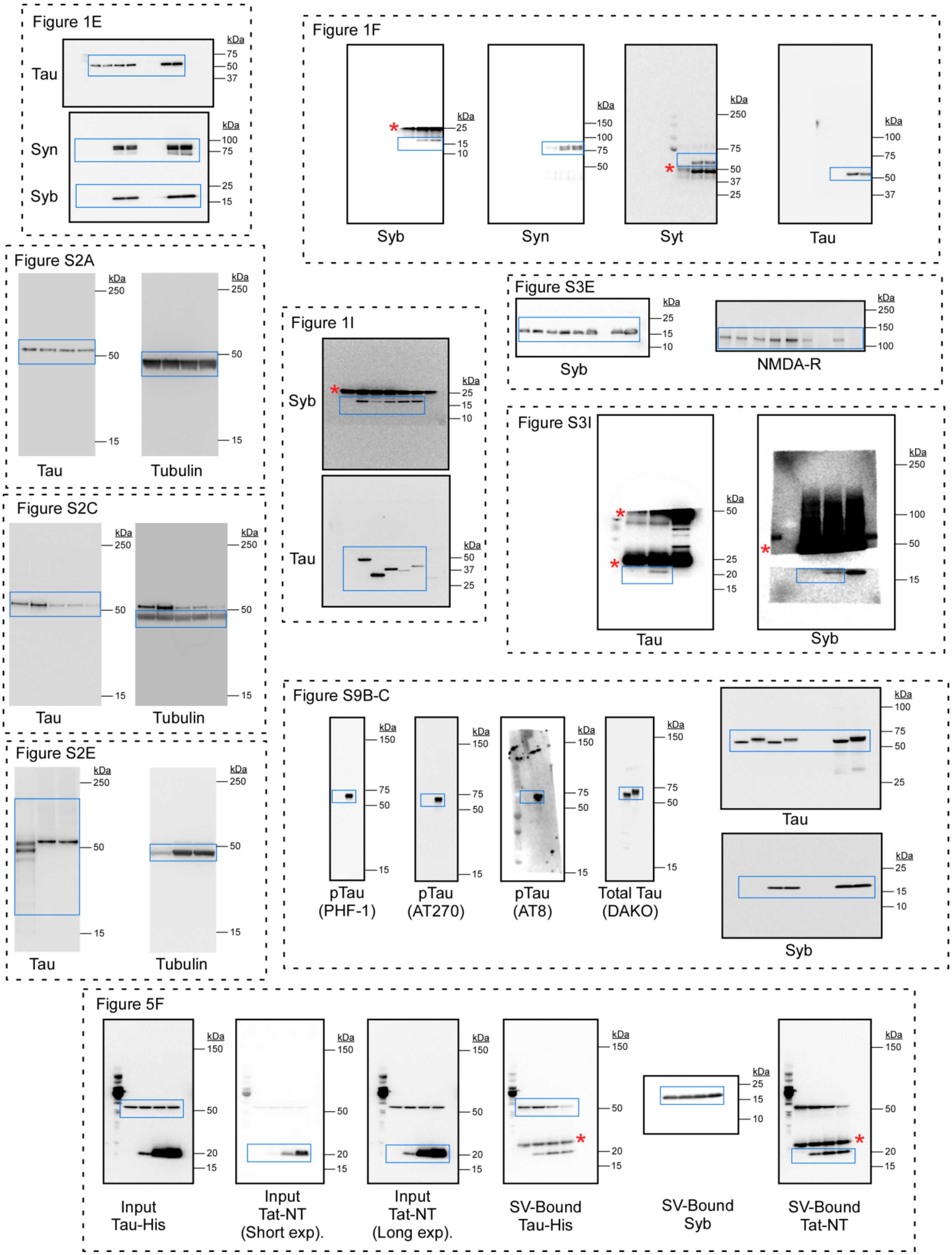

Figure 1E
Tau
kDa
75
50
37
Syn
Syb
kDa
100
75
25
15
Figure 1F
Syb
Syn
Syt
Tau
kDa
25
15
10
kDa
150
100
75
50
kDa
250
75
50
37
25
kDa
100
75
50
37
Figure S2A
Tau
Tubulin
kDa
250
50
15
Figure 1I
Syb
Tau
kDa
25
15
10
kDa
50
37
25
Figure S3E
Syb
NMDA-R
kDa
25
15
10
kDa
250
150
100
Figure S3I
Tau
Syb
kDa
50
25
20
15
kDa
250
100
50
15
Figure S2C
Tau
Tubulin
kDa
250
50
15
Figure S9B-C
pTau (PHF-1)
pTau (AT270)
pTau (AT8)
Total Tau (DAKO)
kDa
150
75
50
15
Tau
kDa
100
75
50
25
Syb
kDa
25
15
10
Figure S2E
Tau
Tubulin
kDa
250
50
15
Figure 5F
Input Tau-His
Input Tat-NT (Short exp).
Input Tat-NT (Long exp).
SV-Bound Tau-His
SV-Bound Syb
SV-Bound Tat-NT
kDa
150
50
20
15
kDa
25
15
10

Supplementary Figure 17. Uncropped immunoblot exposures from immunoblot images displayed in this article. Molecular weight ladders depict separation range. Blue boxes represent cropping as shown in figures. Red asterisks indicate IgG heavy/light chains for immunoprecipitation experiments. Where full separation range is not shown, membranes were physically cut following transfer and immunoblotted for with different antibodies.

Supplementary Table 1. Case information for human brain samples. Related to Supplementary Figure 1.

Case ID	Age	Gender	Diagnosis
AD1	57	Male	AD (Braak VI)
AD2	80	Female	AD (Braak VI)
AD3	90	Male	AD (Braak VI)
Control 1	77	Female	Non-demented Control
Control 2	66	Male	Non-demented Control
Control 3	75	Male	Non-demented Control
Control 4	95	Male	Non-demented Control
